# Supplementary material for: Escalation-related decision making in acute deterioration: a retrospective case note review
Source: BMJ Open. 2018 Aug 17;8(8):e022021. doi: 10.1136/bmjopen-2018-022021 (PMC6104759; doi:10.1136/bmjopen-2018-022021)
Supplement: Supplementary data [file bmjopen-2018-022021supp001.pdf]

Data Extraction Form

**ID number**	
**Age**	
**Gender**	
**Admitting specialty**	
**Date of admission**	
**Date of death**	
**Co-morbidities (from past medical history admission clerking)**	
**History of presenting admission**	
**Cause of death (1a)**	
**Bottom line cause of death (1b or 1c)**	
**Functional status**	
**Potentially avoidable EOLC admission?**	
**Failed EOLC discharge (from evidence in notes)?**	
**Referred to coroner**	
**EOLC pathway used (which and date commenced)?**	
**Was CPR attempted (date)?**	
**Presence of DNACPR (date, signed by, reasons for)?**	
**Is there reference to an advanced care plan?**	

**Date**	Time	Event leading to decision/ discussion (including details surrounding event)	Who recognised/responded to (or led the) event	Speciality (of individual who recognised/responded to event)	Action	Detail about action	Outcome (escalation)	Additional detail around escalation outcome	Outcome (de-escalation)	Additional detail around de-escalation outcome	Involvement of patient and family	Were preferences of patient known in advance?	Detail of discussion with patient/family, including how preferences of others (patient, family, professionals) were taken into account	Details of ward move	Comments
**Line break denotes new episode of deterioration**															
