# Supplementary material for: Escalation-related decision making in acute deterioration: a retrospective case note review
Source: BMJ Open. 2018 Aug 17;8(8):e022021. doi: 10.1136/bmjopen-2018-022021 (PMC6104759; doi:10.1136/bmjopen-2018-022021)
Supplement: Supplementary data [file bmjopen-2018-022021supp002.pdf]

Exemplar Case Studies for the Trajectories[1]

Trajectory 1 Exemplar Case	*Trajectory 2 Exemplar Case*
A 78 year old woman was admitted with reduced consciousness and right sided weakness. She had a past medical history of atrial fibrillation and hypertension, and lived with her daughter. On admission to the emergency department, a chest x-ray, ECG and brain CT were performed, and IV fluids commenced. The decision for a ward-based ceiling of care and DNACPR order was made and subsequently discussed with her daughter, before she was transferred to the acute stroke unit. The following day she was reviewed by the stroke consultant. She showed no signs of improvement; her coma scores remained low and she had clinical signs consistent with a large left middle cerebral artery stroke, which was confirmed by brain CT imaging. Her consultant and clinical team felt that she was unlikely to have any meaningful recovery from her significant brain injury, and the priority of future care should be comfort. A discussion with her family outlined the severity of the stroke, explaining that her condition had not improved in the past 24 hours and that she was unlikely to survive this event. Her family understood the situation and agreed with a palliative approach to care, the consultant emphasizing that she would be kept comfortable. Following this family discussion, a care plan for end of life care was developed and as required palliative medications prescribed. All blood tests, IV fluids and early warning of deterioration scoring ceased. She died peacefully the following day with her family present.	A 76 year old widower, living alone, was admitted due to recent episodes of severe chest pain (both the previous night and reoccurring on the morning of the admission). He had a self-reported history of well controlled non-insulin dependent diabetes. On arrival in accident and emergency he had continued pain, vomiting and ST-segment elevation on ECG. Following consultation with the cardiology consultant and review of the ECG, antiplatelet medications were given and he was taken to the cardiac catheter laboratory for immediate angioplasty. Balloon angioplasty revealed a myocardial infarction, with moderate triple vessel disease. Following angioplasty the patient was transferred to coronary care high dependency, with the aim of discharge after 72 hours if mobilising and pain free.<br><br>On day 2 the patient mobilised with physiotherapy input and was transferred to the cardiac ward. A repeat bedside echocardiogram revealed good left ventricular function. Day 3 the patient continued to improve and discharge was planned for the following day, supported by his family. In the early hours of day 4 an arrest call was made. Cardiopulmonary resuscitation was performed for a suspected pulmonary embolism but the patient remained in asystole throughout and after 30 minutes a consensus decision by the treating clinicians was made to stop.

Key:

DNACPR – do not attempt cardiopulmonary resuscitation
CCF - congestive cardiac failure
COPD – chronic obstructive pulmonary disease
CT – computerised tomography
ECG - electrocardiogram
IV - intravenous

[1] To ensure confidentiality and anonymity details have been changed and the exemplars generated using features across cases within each type

Trajectory 3 Exemplar Case	***Trajectory 4 Exemplar Case***
A 79 year old man was admitted with hip pain and reduced mobility following a fall. He had a past medical history of prostate cancer (treated with a radical prostatectomy over 5 years ago), non-insulin dependent diabetes mellitus, chronic kidney disease, ischaemic heart disease, asthma and depression. He was independently mobile with a frame and received carer visits twice daily at home.<br><br>On admission to the emergency department hip X-ray, blood tests and ECG were performed, and IV fluids commenced. His left leg was shortened and externally rotated, and X-rays confirmed a fractured neck of femur. A hemiarthroplasty was performed the following day and he returned to the ward under the care of the orthogeriatric team.<br><br>Over the following days, he received treatment (IV fluids, diuretics and catheterisation) for acute kidney injury and fluid balance issues, as well as ongoing physiotherapy. On day 13 of admission he became tachycardic and hypotensive, triggering an early warning score and review by his medical team. Investigations including a chest X-ray, dipstick urinalysis, ECG and blood tests revealed a likely urinary tract infection, and oral antibiotics were commenced. After 12 hours with no improvement, his antibiotics were switched to IV route and IV fluids were re-commenced. Despite a further 12 hours of antibiotics, his condition continued to deteriorate with spiking fevers and increasing inflammatory markers, reduced urine output and hypotension. Blood cultures were sent, and a decision was made at this point, conveyed to his family, that he should not be for resuscitation or high dependency/intensive care, due to his significant deterioration despite treatment, and his multiple comorbidities.<br><br>Over the weekend, early warning scores prompted junior doctor reviews and a switch of IV antibiotics. Despite this, the patient was agitated and restless. After review by the on-call registrar, it was felt that he should receive symptomatic treatment only, as despite over 72 hours of IV antibiotics he continued to deteriorate, and was now showing signs of distress. A phone call was made to his next of kin to explain that despite treatment, he had progressively deteriorated due to urinary sepsis, his next of kin agreed that supportive care and symptom control were in his best interests and agreed with a DNACPR decision.<br><br>All unnecessary medications, blood tests and observations were ceased. A referral was made to the palliative care team and symptomatic palliative care medications prescribed. He died two days later.	An 88 year old care home resident was admitted to hospital due to an unwitnessed fall during which a head injury was sustained (whilst on warfarin). He had a previous admission to another hospital within the last month also due to a fall, where his diuretic dose was reduced. On admission he had a variable level of consciousness and hypotension. His nursing home stated that he was normally coherent but had experienced a month of reduced eating and drinking. His co-morbidities were noted as CCF, angina, atrial fibrillation, COPD and dementia. His presenting diagnoses were thought to be due to an infective exacerbation of COPD (hospital acquired pneumonia due to the previous admission) and acute kidney injury due to his poor oral intake over the last month.<br><br>Within 24 hours of admission a DNACPR order was signed by the consultant, in consultation with the patient's son due to the underlying CCF and advanced frailty. It was also decided that treatment should occur at ward level, with intubation and ventilation not being appropriate. Over the next couple of days brain CT scan revealed no cause for the reduced consciousness and he improved clinically with first line IV antibiotics and therapy input from physiotherapy and dietetics.<br><br>By day 6 his delirium continued to improve but there were continuing discussions with his son to highlight that although his father had responded to treatment of the infection the underlying conditions meant that he was unlikely to return to the care home. On days seven and eight activation of early warning scores demonstrated low blood pressure, raised respiratory effort and low oxygen saturation levels. Blood tests and a chest X-ray demonstrated left lung consolidation. The IV antibiotics were switched.<br><br>Days eight and nine saw a fall in the consciousness level of the patient despite treatment. The son was called and it was explained that despite treatment his father had developed multi-organ failure. It was agreed that an individualised end of life care plan should be developed focusing on palliation (regular medications and vital signs recordings were stopped) and a referral was made to the palliative care team. On day 10 this team reviewed the patient who was unresponsive but comfortable and pain free. Palliative care medications were administered as required and the patient died with family by his side 14 days after admission.
