# Supplementary material for: Escalation-related decision making in acute deterioration: a retrospective case note review
Source: BMJ Open. 2018 Aug 17;8(8):e022021. doi: 10.1136/bmjopen-2018-022021 (PMC6104759; doi:10.1136/bmjopen-2018-022021)
Supplement: Supplementary data [file bmjopen-2018-022021supp003.pdf]

Summary of Comments from Expert Clinician Review

**Generic**	• The median ages show that we are dealing with a generally elderly population, but not exclusively<br>• The data show that these patients have a lot of co-morbidity<br>• Multiple team involvement emphasised<br>• Clinician hierarchy is important<br>• Decision-making is much more difficult out-of-hours, e.g. between a Consultant who knows the patient/family/case and an on-call Consultant who does not<br>• The more experienced you become as a clinician, the more you recognise uncertainty. The increased exposure to the "unexpected" (in relation to unexpected outcomes of continuing or withdrawing treatment) means that uncertainty remains high<br>• Time limited escalation decisions (e.g. 24-48hrs of ventilation then stop) are more complex and clinicians do not always stick to them. Ceiling of care decisions (e.g. for non-invasive ventilation/not for ventilation) are less complex<br>• Unexpected deterioration will always occur and is impossible to plan for<br>• Lack of escalation related planning usually due to lack of time, lack of "engagement" (with family) and lack of senior re-evaluation of patients over admission course. There are often differences in opinion regarding the reversibility of issues, and differences in opinion regarding pre-admission co-morbidities (and particularly with next of kin)<br>• Increasing culture of "unrealistic" patient/family expectations. These are rare, but can steer decision-making, it is often the more "distant" family members who are not involved all the way through care
**Trajectory 1**	• The catastrophic event occurs at home prior to admission (in contrast to trajectory 3 where the significant point occurs in the hospital)<br>• Typically represents patients with intracranial bleeds, however there is still some uncertainty. Over time with medical developments, the goalposts move with these types of patients
**Trajectory 2**	• There may be 2 subsets of this type: a – those for whom everything is done, but they still die; b – who improve and then there is an event which catches them (e.g. fall, pneumonia)<br>• Does the cardiopulmonary resuscitation reflect less adequate decision-making?
**Trajectory 3**	• The most common trajectory seen in hospital. De-escalation is staged and there may be "bargaining" with families e.g. not everything that team wished has been achieved as a result of an intervention, therefore may agree to continue with status quo for a further 48 hours for example. Deterioration in current condition – the significant point here is greyer and it is harder to make decisions when considering patients "stuck" on high levels of treatment<br>• Missed opportunities in having de-escalation discussion. Frequent continuation of IV fluids and antibiotics with end of life care, as well as a lack of recognition of the dying phase. Due to so many teams being involved and multiple clinicians, so many differences in opinion/views as to when to discuss<br>• New clinical team/out-of-hours input – this is the most uncomfortable in terms of a "trigger"<br>• Outreach teams often see "insidious decline" that the primary care team do not always recognise
**Trajectory 4**	• Significant amount on on-going investigations/treatment despite early limits (ward level care and DNACPR)<br>• The group that would benefit most from formalised TEPs, as potentially the conversations could be had prior to them being admitted to hospital, either in the GP's surgery, care home or hospital outpatients department

**Recommendations for practice**	• Whole cultural shift needed so that individuals are not frightened to talk about what happens when we get ill. Need to create opportunities for discussion outside of crisis situations<br>• Initial discussion with patients/family at or soon after the point of admission is best practice and important in setting expectations in all trajectories<br>• Triggers for escalation related decision-making should be: admission, first senior review, first review by "usual" clinician (if relevant) and any point of deterioration<br>• Earlier and definitive decision-making required (especially trajectory 3 - new clinical team or out-of-hours input as trigger), but decision-making must be accurate, therefore re-evaluation by seniors is key<br>• Significant dependency (especially pertinent to trajectory 4) is important to capture in notes and history taking, should be a trigger for a formalised TEP<br>• Visiting teams may initiate discussions and decisions but these should be implemented by the team with "ownership" who ideally know the patient best<br>• Need for clarity of decisions and what these mean at a practical level e.g. treatment within the ward environment with IV fluids and antibiotics etc.<br>• Need for clarity of terminology and meaning in practice. Palliation means different things to different people and is often a source of confusion. Palliative treatment and full escalation including CPR and ITU are not mutually exclusive. "Ward-based care" is used frequently, meaning potentially aggressive treatment up to the limits of what is possible in a non-HDU and ITU setting (and DNACPR) but it can be misinterpreted in practice as effectively meaning end of life care
