# Supplementary material for: Escalation-related decision making in acute deterioration: a retrospective case note review
Source: BMJ Open. 2018 Aug 17;8(8):e022021. doi: 10.1136/bmjopen-2018-022021 (PMC6104759; doi:10.1136/bmjopen-2018-022021)
Supplement: Author's manuscript [file bmjopen-2018-022021.draft_revisions.pdf]

BMJ Open is committed to open peer review. As part of this commitment we make the peer review history of every article we publish publicly available.

When an article is published we post the peer reviewers' comments and the authors' responses online. We also post the versions of the paper that were used during peer review. These are the versions that the peer review comments apply to.

The versions of the paper that follow are the versions that were submitted during the peer review process. They are not the versions of record or the final published versions. They should not be cited or distributed as the published version of this manuscript.

BMJ Open is an open access journal and the full, final, typeset and author-corrected version of record of the manuscript is available on our site with no access controls, subscription charges or pay-per-view fees (http://bmjopen.bmj.com).

If you have any questions on BMJ Open's open peer review process please email info.bmjopen@bmj.com

BMJ Open

ESCALATION RELATED DECISION-MAKING IN ACUTE DETERIORATION: A RETROSPECTIVE CASE NOTE REVIEW

Journal:	*BMJ Open*
Manuscript ID	bmjopen-2018-022021
Article Type:	Research
Date Submitted by the Author:	30-Jan-2018
Complete List of Authors:	Campling, Natasha; University of Southampton Faculty of Health Sciences,<br>Cummings, Amanda; University of Southampton, Faculty of Health Sciences<br>Myall, Michelle; University of Southampton, Faculty of Health Sciences<br>Lund, Susi; University of Southampton, Fcaulty of Health Sciences<br>May, Carl; University of Southampton, Faculty of Health Sciences<br>Pearce, Neil; University Hospital Southampton NHS Foundation Trust<br>Richardson, Alison; University of Southampton, Faculty of Health Sciences
Keywords:	decision-making, treatment escalation, de-escalation, palliation, resuscitation, goals of care

SCHOLARONE™
Manuscripts

For peer review only

ESCALATION RELATED DECISION-MAKING IN ACUTE DETERIORATION: A RETROSPECTIVE CASE NOTE REVIEW

Natasha Campling[1,2], Amanda Cummings[1,2], Michelle Myall[1,2], Susi Lund[1,2], Carl R. May[1,2,3], Neil W. Pearce[3], Alison Richardson[1,2,3]

1. Faculty of Health Sciences, University of Southampton, UK
2. NIHR CLAHRC Wessex, University of Southampton, UK
3. University Hospital Southampton NHS Foundation Trust, Southampton, UK

Corresponding author:
Dr Natasha Campling, Faculty of Health Sciences, University of Southampton, Building 67, University Road, Highfield, Southampton, SO17 1BJ
Tel: +44 (0)23 8059 8228; Fax +44 (0)23 8059 8308; Email: n.c.campling@soton.ac.uk

Key words:
Decision-making; treatment escalation; de-escalation; palliation; resuscitation; goals of care

Word count: 4,966 (excluding abstract, references and tables)

For peer review only

For peer review only - http://bmjopen.bmj.com/site/about/guidelines.xhtml

ABSTRACT (252 words)

Aim: To describe how decision-making interrelates with the sequence of events in individuals who die during admission; and identify situations where formal treatment escalation plans (TEPs) may have utility.

Design and methods: A retrospective case note review, utilising stratified sampling. Two data analysis methods were applied concurrently: directed content analysis; and care management process mapping via annotated timelines for each case. Analysis was followed by expert clinician review (n=7), contributing to data interpretation.

Sample: 45 cases, age range 38-96 years, 23 females and 22 males. Length of admission ranged from <24hours to 97 days.

Results: Process mapping led to a typology of care management, encompassing four trajectories: early de-escalation due to catastrophic event; treatment with curative intent throughout; treatment with curative intent until significant point; and early treatment limits set. Directed content analysis revealed a number of contextual issues influencing decision-making. Three categories were identified: multiple clinician involvement, family involvement, and lack of planning clarity; all framed by clinical complexity and uncertainty.

Conclusions: The review highlighted the complex care management and related decision-making processes for individuals who face acute deterioration. These processes involved multiple clinicians, from numerous specialities, often within hierarchical teams. The review identified the need for visible and clear management plans, in spite of the frame of clinical uncertainty. Formal TEPs can be used to convey such a set of plans. Opportunities need to be created for individuals to request these be developed, in consultation with the clinicians who know them best, out with the traumatic circumstances of acute deterioration.

Strengths and limitations of this study

- There is a lack of description of escalation-related decision-making in the context of deterioration outside of the critical care environment. Our study setting was the comprehensive hospital environment, and individuals who were facing acute deterioration that led to death.
- The study explored clinical decision-making processes: the types and range of decisions made, the involvement of families in these processes, and the interaction between clinical teams. Care management trajectories provoked by acute deterioration were characterised, via typology; including points of significance in the sequence of events.
- While the sample was stratified it was small, selected from a single acute hospital Trust. However two data analysis methods were applied concurrently (followed by expert clinician review): directed content analysis; and care management process mapping via annotated timelines.
- Examination of decision-making processes highlighted areas for improvement and the potential impact of formal treatment escalation plans through pre-emptive decision-making and patient involvement out with crisis situations.

BACKGROUND

Clinical decision-making in the context of acute deterioration during hospital admission is complex. Such decisions are frequently made in the face of uncertainty, characterised by: lack of underpinning information or diagnostic clarity, necessity for rapid decision-making, and the inability of patients to collaborate in discussions and decisions because of the acuity of their condition [1].

Previous research has focused on the illness trajectories of deteriorating patients, or on clinical decision-making in the specific context of critical care. The wider context of care management and

For peer review only - http://bmjopen.bmj.com/site/about/guidelines.xhtml

related decision-making remains an un-researched area. Murray et al (2005) highlighted the value of awareness of illness trajectories as a mechanism for clinicians to help plan care to meet patients' needs and for families to cope [2]. More recently, Etkind and colleagues (2014) defined trajectories of final illness among patients who died whilst inpatients [3]. These were defined *a priori* to their case-note review as: predictable (gradual deterioration during admission); predictable (rapid deterioration during admissions); unpredictable course during hospital admission; and sudden death. 149 cases were examined (all deaths over 11 months on five inpatient wards where the AMBER care bundle was implemented) and characterised according to one of four trajectories.

Our study, progresses the above which focused exclusively on illness trajectories, by expanding the focus to care management and understanding of the associated decision-making processes, to inform clinical practice. Higginson and colleagues (2016) explored this area by examining patterns of decision-making, but their work was specific to critical care [4]. Only 16 cases were examined (in combination with interviews and non-participant observation), and four trajectories with different patterns of clinical decision-making identified: curative care from admission (to critical care); oscillating curative and comfort care; shift to comfort care; and comfort care from admission. They emphasised that "conflict" in decision-making could occur, between relatives and staff and between and within clinical teams.

Given the complexity of clinical decision-making related to acuity and deterioration, and perhaps the likelihood for "conflict" [4], there has been a move nationally and internationally to develop and implement formal treatment escalation plans (TEPs). Structured, procedure specific TEPs are proposed as a mechanism by which to improve understanding and communication when escalation related decisions need to be made and acted on [5]. They provide a framework on which to base a conversation and document treatment options that are appropriate if a patient were to become acutely unwell. They vary in both design and use [5,6,7]. Notable examples include: Universal Form of Treatment Options (UFTO) [8], Deciding Right http://www.nescn.nhs.uk/common-themes/deciding-right/ and Physician Orders for Life Sustaining Treatment (POLST) [9]. In the UK, there is growing interest in the national initiative, instigated in 2014, led by the Resuscitation Council and the Royal College of Nursing which generated the Recommended Summary Plan for Emergency Care and Treatment (ReSPECT) http://www.respectprocess.org.uk/

Despite this increasing awareness, little work has examined the implementation of TEPs. This study is part of a wider programme of work to inform the implementation and evaluation of TEPs as part of the *Complexity, Patient Experience and Organisational Behaviour* theme of the NIHR Collaboration for Applied Health Research and Care Wessex (NIHR CLAHRC Wessex). Additionally, the team have undertaken a review of communication and decision-making interventions directed at goals of care, via a theory led scoping review [10].

AIMS/OBJECTIVES

The study explored the care management of those who deteriorate and die during hospital admission, characterising the resources mobilised, in as much detail as could be tracked through recourse to case notes. The aims were: to describe how decision-making processes interrelate with the sequence of events for individuals who die during inpatient admission; and to identify situations where treatment escalation plans may have had utility.

The objectives were:

For peer review only - http://bmjopen.bmj.com/site/about/guidelines.xhtml

1. To identify and characterise, via the generation of a typology, the care management trajectories of hospital inpatients facing acute pathophysiological deterioration which ultimately leads to death.
2. To map clinical decision-making processes, including the involvement of patients and families in decisions, identifying what leads to and triggers changes in management.
3. To identify the potential role of treatment escalation plans in providing a framework to support discussions and recording of decisions.

METHODOLOGY & METHODS

Study Design

A retrospective case note review, exploring the care management of those who die during hospital admission.

Sampling Strategy

The case note review followed an initial audit of death certificate review forms (DCRFs) from all deaths at a single acute hospital Trust in England (n=911) within a six month period (January-July 2015). Case notes of a 5% sample (45 sets of notes) of patients, aged over 18 were reviewed. The DCRF data enabled stratified sampling, ensuring appropriate representation across groups. 32 mutually exclusive strata were created based on whether or not cases had all possible combinations of the following: DNACPR, palliative care team involvement, intensive care/high dependency management, evidence of escalation/de-escalation decision, and unpredictable illness trajectory [3]. Proportionate allocation was used to sample the same fraction from each strata, with a check that the total sample size was calculated as expected (i.e. not affected by rounding of the numbers for each strata to integers).

Data Collection

Data were collected from case notes only. Data collection tracked the period from admission to hospital through to each patient's death to identify: 1) when decisions to escalate or de-escalate treatment were made, 2) how those decisions were made, and 3) who was involved in these decisions. For those with a prolonged admission (> 30 days), data collection was limited to the last 30 days of admission (but included social and clinical data regarding their admission). The following data were extracted:

- Clinical and demographic information regarding admission to hospital, including but not limited to, comorbidities and admitting specialism.
- End of life care and DNACPR information, including but not limited to, whether CPR was attempted.
- Nature of any events leading to a discussion or decision regarding levels of care, who recognised and responded to the event, actions taken, further detail on escalation or de-escalation of care and outcomes from this. Here, "event" referred to episodes such as clinical deterioration, ward rounds, specialist review or emergence of new clinical findings.
- How decisions were documented, including clarity of documentation and use of care plans.
- Evidence of patient and/or family involvement in decision-making and how patient preferences and those of others are taken into account, including whether patient wishes were known in advance.
- Ward movements.
- Date and cause of death.

For peer review only - http://bmjopen.bmj.com/site/about/guidelines.xhtml

Data extraction was undertaken by two clinically qualified researchers (NC & AC) and data recorded using an Excel spreadsheet pro forma (see supplementary file 1). The pro forma was piloted on a set of notes and based on this changes to the form were made to facilitate usability and increase reliability of data extraction. This resulted in: inclusion of all causes of death (not just cause 1a but also underlying causes 1b and 1c), and enabling of free text entry for avoidable end of life care (EOLC) admission and failed EOLC discharge. The revised pro forma was tested by NC and AC on an initial sample of case notes (n=8) to assess utility and consistency of data entry. No further changes were required. At the end of data collection a process of cross-checking by both researchers helped to mitigate against errors, ensure accuracy and consistency.

Data Analysis

Two methods of data analysis were applied concurrently. Firstly, case notes were treated as qualitative data and analysed using directed content analysis [11]. The data within the pro forma were analysed using this method and directed towards: the event leading to the decision or discussion; and the action taken and resulting outcomes, and details regarding involvement and discussion with the patient and family. Data comprised verbatim transcription of relevant entries in the case notes to the pro forma. Additionally, field notes were analysed to capture limitations of case notes as a data source and recurrent issues (sequence of events and triggers for decision-making) across cases.

Secondly, care management process mapping via annotated timelines involving key events were developed for each case [12]. These timelines included: escalation and de-escalation related decisions; involvement of patient and family in decision-making; clinical treatment plans made; investigations undertaken and treatment received; and key clinical information to inform probability of outcomes and prompt decisions.

Timelines were drawn for each case (NC & AC) and then grouped independently by NC & AC (double screened) into one of four care management trajectories which became apparent during analysis. Categorisation of cases by the researchers were compared, with input from two additional clinical members of the research team (SL & AR). Where there was initial disagreement, the pro formas were revisited in a team discussion to agree final categorisation (n=11).

Diagrams were subsequently drawn to represent the group experience of the four care management trajectories. These were iteratively refined (NC, AC, SL & AR). They were combined with tabulated data representing the cases within each trajectory and a case exemplar (case study) and sent to a group of expert clinicians (representing a wide range of specialities) for review. They were asked to consider:

- Do the 4 care management trajectories capture the sequence of events and decision-making processes involved?
- Do the trajectories apply to patients you have seen recently who have then gone on to die whilst in hospital? Could you consider how they do or do not apply?
- Do these data demonstrate potential triggers for decision-making or treatment escalation planning that you would like to see put into practice?
- Is there anything in the data you are surprised by or any other comments you would like to make?

Out of 13 experts approached, 7 commented in detail on the data either face to face or via telephone/email. Their feedback verified: the care management trajectories reflected what

For peer review only - http://bmjopen.bmj.com/site/about/guidelines.xhtml

clinicians encounter in practice and were described in a way they could identify with; the classification of cases to the trajectories; and the authenticity of the case exemplars. They contributed to the overall interpretation of data.

Ethical and Research Governance Considerations

Ethical approval (via the Health Research Authority and Research Ethics Committee South Central – Hampshire A ref: 16/SC/0599) and research governance approval were gained for the study. As access to patient identifiable data (case notes) was required without consent, support under section 251 of the NHS Act (2006) was sought and obtained via the Health Research Authority's Confidentiality Advisory Group [13].

Patient and Public Involvement

A PPI champion worked closely with the research team on this study, and the wider programme, informing all study processes. Involvement led to the recommendation that the team solely access paper based notes (to restrict the amount of data accessed) and not electronic medical records as originally planned.

RESULTS

Sample characteristics

The age range of patients included (in the review of the 45 sets of notes) was 38-96 years, with 23 female and 22 male. The length of admission ranged from <24hours to 97 days. Thirty five patients had a DNACPR in place at time of death. Fifteen patients had palliative care team involvement.

A Typology of Care Management

Analysis via process mapping led to the development of a typology of care management, encompassing four distinct trajectories. The trajectories characterised the sequence of events and decision-making processes through acute pathophysiological deterioration leading to death. They were:

1. Early de-escalation (within 24-48 hours of admission) due to catastrophic event - clinically observable signs and symptoms +/- observable on imaging
2. Treatment with curative intent throughout (no de-escalation) +/- cardiopulmonary resuscitation
3. Treatment with curative intent until significant point
4. Early treatment limits set (within 48 hours of admission)

Table 1 displays the key characteristics of the cases represented by each trajectory. Each care management trajectory is described in sequence below, including a diagrammatic representation of the respective trajectory. Exemplar case studies for each trajectory are included in supplementary file 2. The process of reviewing the data with expert clinicians added a valuable dimension to data interpretation. The depth and range of their feedback, via their experiential knowledge, is summarised in supplementary file 3.

For peer review only - http://bmjopen.bmj.com/site/about/guidelines.xhtml

Table 1 - Key Characteristics of Cases within the Trajectories

TRAJECTORY 1 CASES (n=10)			TRAJECTORY 2 CASES (n=8)		
**AGE (MEDIAN, RANGE)**	79.5 (47-94) years		**AGE (MEDIAN, RANGE)**	83 (53-96) years	
**GENDER**	6 female; 4 male		**GENDER**	1 female; 7 male	
**CACI[1] COMORBIDITIY SCORE (MEDIAN, RANGE)**	5.5 (4-10)		**CACI COMORBIDITIY SCORE (MEDIAN, RANGE)**	6.5 (2-9)	
**SOCIAL HISTORY**	Care/nursing home resident or respite<br>Home carers	2<br>1	**SOCIAL HISTORY**	Care/nursing home resident or respite<br>Home carers	1<br>2
**LENGTH OF ADMISSION (MEDIAN, RANGE)**	3 (1-16[2]) days		**LENGTH OF ADMISSION (MEDIAN, RANGE)**	7.5 (2-19) days	
**PRIMARY REASON FOR ADMISSION**	Cerebrovascular accident<br>Gastrointestinal<br>Sepsis<br>Ischaemic cardiac disease<br>Peripheral vascular disease	3<br>2<br>3<br>1<br>1	**PRIMARY REASON FOR ADMISSION**	Respiratory (infective)<br>Ischaemic/arrhythmic cardiac disease<br>Fall<br>Fracture<br>Cellulitis	3<br>2<br>1<br>1<br>1
**TRIGGERS FOR RECOGNITION OF IRREVERSIBILITY/ UNSURVIVABLE EVENT**<br>*SOME CASES HAD MORE THAN ONE TRIGGER	Imaging results<br>Clinically observable diagnosis<br>Consultant review<br>Reduced consciousness	3<br>4<br>1<br>3	**ONGOING CARE MANAGEMENT/ TREATMENT ISSUES**<br>*ALL CASES HAD MULTIPLE ISSUES	Fluid balance (cardio-renal failure)<br>Acute (on chronic) kidney injury<br>Ischaemic/arrhythmic cardiac disease<br>Respiratory tract infection<br>Urinary tract infection<br>Diabetic control<br>Pulmonary embolism<br>Respiratory failure	4<br>2<br>5<br>8<br>1<br>2<br>1<br>4
**RECEIVED CCO/ITU[3] REVIEW**		1	**RECEIVED CCO/ITU REVIEW**		1
**RECEIVED HDU/ITU[4] CARE**	Intensive care unit	1	**RECEIVED HDU/ITU CARE**	High dependency unit<br>Intensive care unit	1<br>1
			**CPR[5] ATTEMPTED AND UNSUCCESSFUL**		5
			**REASONS FOR NO DE-ESCALATION**<br>*SOME CASES HAD MORE THAN ONE REASON	Awaiting transfer/discharge<br>Patient preference/limited or no family involvement<br>Young/normally fit and well/few comorbidities<br>Post (curative intent) intervention<br>Input from multiple specialist teams	3<br>2<br><br>3<br>1<br>2

For peer review only - http://bmjopen.bmj.com/site/about/guidelines.xhtml

TRAJECTORY 3 CASES (n=18)			TRAJECTORY 4 CASES (n=9)		
**AGE (MEDIAN, RANGE)**	80.5 (38-88) years		**AGE (MEDIAN, RANGE)**	86 (63-91) years	
**GENDER**	12 female; 6 male		**GENDER**	4 female; 5 male	
**CACI COMORBIDITIY SCORE (MEDIAN, RANGE)**	6 (1-12)		**CACI COMORBIDITIY SCORE (MEDIAN, RANGE)**	8 (5-14)	
**SOCIAL HISTORY**	Care/nursing home resident or respite<br>Home carers	1<br>4	**SOCIAL HISTORY**	Care/nursing home resident or respite<br>Home carers	5<br>1
**LENGTH OF ADMISSION (MEDIAN, RANGE)**	17.5 (3-97) days		**LENGTH OF ADMISSION (MEDIAN, RANGE)**	12 (2-28) days	
**PRIMARY REASON FOR ADMISSION**	Gastrointestinal<br>Cerebrovascular accident<br>Respiratory tract infection<br>Urinary tract infection<br>Specialist treatment (chemotherapy, cardio ablation)<br>Haematological<br>Fracture<br>General decline + hypertension<br>Respiratory	3<br>2<br>5<br>1<br>2<br>1<br>1<br>2<br>1	**PRIMARY REASON FOR ADMISSION**	Sepsis<br>Respiratory tract infection<br>Malignancy<br>Cerebrovascular accident<br>Fall<br>Acute heart failure	3<br>2<br>1<br>1<br>1<br>1
**RECEIVED CCO/ITU REVIEW**		4	**PRE-EXISITING FACTORS CHARACTERISING ADMISSION**<br>*SOME CASES HAD MORE THAN ONE FACTOR	Frailty<br>History of recent deterioration<br>Pre-existing DNACPR[6]<br>Current malignancy<br>Underlying dementia<br>Already known to palliative care	5<br>2<br>4<br>3<br>3<br>3
**RECEIVED HDU/ITU CARE**	Intensive care unit	3	**PROMPTS FOR SETTING EARLY TREATMENT LIMITS**<br>*SOME CASES HAD MORE THAN ONE PROMPT	Senior clinician review<br>Patient's prior wishes expressed by family<br>Discussion with patient<br>Marked deterioration	7<br>4<br>3<br>4
**SIGNIFICANT POINT TRIGGERING DE-ESCALATION**<br>*SOME CASES HAD MORE THAN ONE TRIGGER	Significant deterioration in current condition<br>New diagnosis leading to deterioration in condition<br>New clinical team/out of hours input recognising poor prognosis	6<br>11<br>4			

Notes:
[1] CACI - Charlson Age Comorbidity Index www.pmidcalc.org/7722560 (Charlson et al 1994); [2] One individual lived for 16 days despite catastrophic event due to younger age; [3] Critical care outreach/intensive care review; [4] High dependency/intensive care; [5] Cardiopulmonary resuscitation; [6] Do not attempt cardiopulmonary resuscitation

For peer review only - http://bmjopen.bmj.com/site/about/guidelines.xhtml

1. Early De-Escalation Due to Catastrophic Event

This trajectory was characterised by hospital admission due to "catastrophic" events (Figure 1). The event had occurred outside of hospital, was evident at the point of admission, and referenced individuals who were in danger of dying on admission (e.g. patients who were moribund secondary to shock), or those admitted with severe, critical illnesses (e.g. major cerebrovascular accidents).

Following admission, there was a period of initial escalation, with accompanying imaging, diagnostic investigations such as blood tests or electrocardiograms (ECGs), and treatment with intravenous (IV) antibiotics or fluids. This escalation also encompassed senior or specialist (e.g. surgical and intensive care) review.

A key feature of this trajectory was the early (within 24-48 hours) recognition of an unsurvivable or irreversible event. All cases had at least one factor that identified this including: imaging results, clinically observable diagnoses, reduced level of consciousness and/or consultant review. Following recognition of futility, discussions with family and next of kin preceded palliation in all cases bar one. In this case, deterioration and death were so rapid as to prevent timely palliation. This trajectory was generally defined by short admissions, on average, patients died within three days.

Figure 1 – Early de-escalation due to catastrophic event

2. Treatment with Curative Intent Throughout

Trajectory 2 was characterised by treatment with curative intent for the duration of hospital admission (Figure 2). Individuals were admitted with a variety of diagnoses, and admissions were characterised by ongoing care at ward or high dependency/intensive care (HDU/ICU) level for multiple issues. These included fluid balance management associated with cardio-renal failure or acute kidney injury, treatment of infections and management of ischaemic or arrhythmic cardiac disease.

This trajectory was also characterised by the development of new diagnoses (e.g. sepsis) or sudden, unpredictable events (e.g. pulmonary embolism) which ultimately led to death. However, in these cases, such events did not trigger de-escalation (as in trajectory 3), patients were actively treated until death. In 5 of the 8 cases unsuccessful cardiopulmonary resuscitation occurred prior to death. In the three remaining individuals, DNACPR orders had been stimulated by senior clinician reviews and/or family discussions.

The reasons underlying a lack of de-escalation related to patient characteristics, individual preferences and the delivery or focus of health care. Some individuals were younger or normally fit and well with minimal comorbidities, whilst others expressed a preference for active treatment. For some, a recent intervention with curative intent, or the fact that they were awaiting discharge or transfer to alternative settings, meant that de-escalation was not a consideration. In others, the involvement of multiple specialist teams meant that the leading specialism (and thus the team who might be expected to make de-escalation decisions) was not clear.

Figure 2 – Treatment with curative intent throughout

3. Treatment with Curative Intent until Significant Point

Trajectory 3 was characterised by curative intent treatment until a significant point, triggering de-escalation of care (Figure 3). These triggers included significant deterioration in the patient's current

For peer review only - http://bmjopen.bmj.com/site/about/guidelines.xhtml

condition (in the absence of a new diagnosis) for example a reduction in consciousness level or patient agitation/distress, and new diagnoses (e.g. infection or malignancy) which led to deterioration in the patient's condition. A third trigger involved a new clinical specialism or out-of-hours review recognising poor prognosis and the futility of current treatment, prompting de-escalation.

All triggers for de-escalation prompted discussions with next of kin, family and the patient or other clinical teams (if under shared-care management). Following these discussions, multi-staged de-escalation ensued. The first stage involved the setting of ceilings of care and DNACPR orders. This first stage at times occurred prior to family discussion, but such discussion always preceded the second stage, which included stopping vital sign observations, early-warning activation scores and invasive investigations/treatments. In some cases, a time and intensity limited trial of treatment (e.g. antibiotics) preceded a third stage of de-escalation, palliation. For patients receiving HDU/ICU level care, the latter stages of de-escalation involved the withdrawal of treatment. There was usually some degree of treatment provided in parallel to multi-staged de-escalation, although this was limited, typically involving antibiotics and IV fluids. The time between the significant point which triggered de-escalation and patient death was between 0-10 days, however this trajectory was characterised by the longest and most varied admission length, 3-97 days.

Figure 3 – Treatment with curative intent until significant point

4. Early Treatment Limits Set

Trajectory 4 was characterised by the presence of early treatment limits, set within 48 hours of admission (Figure 4). The triggers for setting limits included patient refusal of treatment, discussions with family, senior clinician review and marked deterioration in the patients' condition. Crucially, these triggers occurred against backgrounds of: history of recent deterioration, frailty, underlying diagnoses of dementia or malignancy, and the presence of pre-existing DNACPR orders and palliative care involvement. In line with this, the patients in this trajectory had the highest average comorbidity scores and ages.

Early treatment limits formed the start of a multi-staged de-escalation process, which occurred across the duration of admission. This de-escalation started with treatment limits (DNACPR, not for intubation/dialysis/ICU care, ward based care) before progressing to more active de-escalation (ceasing early warning scores, ceasing antibiotics/IV fluids/regular medications, palliation and commencement of an individualised end of life care plan).

Key to this trajectory was the level of on-going treatment in parallel with the staged de-escalation. Despite early treatment limits being set, on-going treatment involved a far more extensive range of treatment (interventions, therapy and medications) than in trajectory 3. Interventions included catheterisation, nasogastric tubes and blood transfusions. There was therapy input from physiotherapists, speech and language therapists, dieticians and occupational therapy teams, and medications included diuretics and antibiotics. Nonetheless, on-going treatment was restricted to a ward environment as clinical history meant these individuals were not candidates for intensive treatment.

Figure 4 – Early treatment limits set

<u>The Categories</u>

For peer review only - http://bmjopen.bmj.com/site/about/guidelines.xhtml

In addition to the care management typology, our directed content analysis revealed a number of contextual issues, which influenced decision-making. We identified three inter-linked categories consisting of: multiple clinician involvement, family involvement, and lack of planning clarity. The categories were framed by clinical complexity and uncertainty.

Clinical Complexity and Uncertainty

The cases demonstrated clinical complexity caused in the main by multiple co-morbidities, new diagnoses or undiagnosed conditions, and challenging management e.g. of sepsis, kidney injury and frailty. Challenging management of fluid balance issues associated with multiple concurrent comorbidities, and the onset of new infections, were a frequent occurrence. A lack of clarity surrounding definitive diagnoses often meant that clinicians were "working in the dark" trying to maximise management despite ongoing uncertainty. Although there were some more clearly defined diagnoses and management paths evidenced (such as stroke), with greater clinical predictability, these cases were in the minority.

Decision-making was complicated by frequently changing clinical situations, particularly in relation to new findings or diagnoses. Escalation-related decisions were required that could adapt to these changing situations, where previous management plans were rapidly rendered inappropriate.

Multiple Clinician Involvement

Clinical management via multiple specialities, therapy and outreach teams, could preclude sight of the patient's prognosis. This was evidenced by treatment decisions and therapy involvement that did not always reflect an individual's prognosis. Likewise, the practicalities of input from multiple specialisms, including numerous repeat reviews and interplay between different teams, often acted to elongate decision-making processes, and added complexity when no-one team took responsibility for leading decisions.

There was evidence of a hierarchy in decision-making, with senior clinicians most often instigating decisions. Junior doctors were less likely to make escalation related decisions, especially concerning placing limitations on, or removal of, treatments. Junior doctors, when required to make decisions alone (particularly those working out-of-hours) were more likely to continue treatment escalation, especially in the absence of pre-specified escalation plans. As such, there was a clear role for senior review, with registrars and consultants instigating the majority of decisions regarding treatment limits and withdrawal of treatment.

The transfer of patients between wards and clinical teams added complexity to decision processes. There was evidence of transfers resulting in de-escalation plans being overlooked, however in other circumstances, ward or team moves prompted new reviews and the initiation of appropriate planning. The positive influence of new perspectives or "fresh eyes" on escalation related decision-making was apparent, especially via out-of-hours clinicians. It appeared that individuals not caught up in the day-to-day management of patient care were able to see the "bigger picture" regarding care management, often initiating ceilings of care, or prompting escalation plans.

Family Involvement

The role and influence of the family was often central in the decision-making process. It was apparent that escalation-related decisions (i.e. whether to continue to increase the intensiveness of treatment e.g. dialysis, intubation and ventilation or maintain treatment at ward level) were often established and actioned before discussions with the family took place. Whereas, de-escalation

For peer review only - http://bmjopen.bmj.com/site/about/guidelines.xhtml

related decisions (such as ceasing treatments and commencing palliation) were postponed until after discussions with family. Family involvement and consensus agreement always preceded the withdrawal of treatment (e.g. organ support and ventilation). This reflects the moral imperative to discuss such decisions with family. Additionally, family were involved in the decision-making process for DNACPR orders where there was any concern about patient competency. Families also played an important role in providing collateral histories for clinicians, enabling decision-making to be placed in the context of an individual's recent health. This was particularly the case with older patients where families could highlight weeks or months of recent deterioration or recurrent infections, aiding the admission clerking, and facilitating early treatment limits being set (trajectory 4).

The impact of the familial role was most apparent when absent. In a few cases, where patients had limited or no family involvement, or lacked the physical presence of family members to prompt discussions, de-escalation decisions were not made (those in trajectory 2). In contrast, where families were engaged they were frequently involved in consultative decision-making with clinical teams. These families were often able to provide clear instructions to clinicians because of their knowledge of patients' prior wishes. For example, relatives were recorded as stating that the "*patient wouldn't want to live like this*", and were therefore more likely to endorse clinician recommendations for treatment withdrawal. Additionally, families often agreed with recommendations that if the patient did not respond to treatment then a move to focus on palliation should occur. The converse did apply, although only in a few cases, whereby families stated that the patient would "*want all done*". In situations where families were unsure of the patient's wishes, further team meetings with the family were always undertaken.

Lack of Planning Clarity

The data revealed a general lack of clarity and visibility regarding management plans in the case notes. However, the clinical complexity of these cases at times precluded the making of escalation related plans or led to them being held in a type of uncommitted management "status" until certainty was gained. Even where cogent management plans were made, they may not have been followed because there were no effective methods for signposting clinicians to plans buried in subsequent pages of notes. In addition, where management plans involved clear de-escalation, these were not always followed. This was sometimes more than just due to the lack of visibility in the notes, but also due to clinical complexity and unpredictability of deterioration, with fluctuations leading to patients temporarily improving or stabilising.

Initial clerking and history taking was paramount to the quality of decision-making throughout admission. This was particularly apparent where clerking histories appeared "lost", with key factors not carrying through into decisions made. Where an important co-morbidity was not acknowledged during the admission clerking, this could continue to influence care over the length of admission.

DISCUSSION

This case note review and qualitative analysis, identified four care management trajectories, defining and mapping clinical decision-making processes in the context of acute pathophysiological deterioration. All trajectories from admission through to death, were framed by clinical complexity and related uncertainty. In general, such complexity confounded decision-making processes. Nonetheless, in a minority of profoundly complex cases (e.g. older age, associated frailty, comorbid and premorbid statuses), complexity could encourage escalation related decision-making. This was apparent in the fourth trajectory, where early treatment limits were set based on patients' pre-

For peer review only - http://bmjopen.bmj.com/site/about/guidelines.xhtml

admission morbidity. This concurs with Fritz et al (2014) who in a retrospective case note review found a lower threshold for completing DNACPR orders in patients with multiple co-morbidities [14].

The trajectories identified here expand those previously described by Higginson et al (2016) which were exclusive to critical care, as they are applicable to hospital inpatients irrespective of care setting [4]. Consequently, our trajectories highlight a) significant points in care trajectories where senior secondary review and re-evaluation of management plans would be valuable, and b) groups of patients for whom a formal TEP would be of particular benefit, as a framework to support discussions and the recording of decisions.

Our findings display significant points in care management trajectories (1&3). These included the recognition of irreversibility, deterioration in current condition, new diagnoses leading to deterioration, and new clinical or out-of-hours team involvement. It was these points that triggered discussions around escalation, and ultimately decision-making. We propose that whilst acting as triggers, these points in trajectory 3 cases also present missed opportunities, for earlier, timely decision-making. It was frequent for deterioration to occur out-of-hours, with important decisions left to on-call teams and sometimes more junior clinicians. As previous studies have shown, this can preclude decisions that reflect the best-interests and preferences of the patient [12]. Here, clear management plans are required that pre-empt the possibility of deterioration and outline the patients' wishes in such circumstances, as well as realistic parameters of care.

The absence of significant points in some cases by which to trigger decision-making, such as those in trajectory 2, leads to a proposition made by the study's expert clinical reviewers that strategic senior reviews are required. It is possible that earlier senior review secondary to a post-admission review, may enable appropriate re-evaluation and alter management plans. Nevertheless, a lack of recognition of the dying phase, even by senior clinicians, highlighted the role and contribution of palliative care teams in questioning ongoing investigations or treatment, and stimulating appropriate symptom control.

It is known that formal TEPs are helpful in stimulating discussions, formulating clear plans, ensuring patient preferences are considered [5,15], and perceived as a good idea by patients, families and healthcare professionals [5,15,16,17]. In addition, they help healthcare professionals structure their discussions with patients and families, and record their decisions, improving documentation clarity [18] and escalation-related communication within clinical teams [16,19]. Despite this, in the case notes reviewed, there were no recorded instances of a formal TEP being used to aid decision-making. Four patients held pre-existing DNACPR orders, but none had evidence of an advance care plan or formal TEP. Despite the small number of pre-existing DNACPR orders in the review, their existence led clinicians to have wider escalation related discussions with patients and families. There is also a pragmatic argument that documenting a DNACPR decision should trigger consideration of a TEP, as a logical continuation of the resuscitation discussion. However, based on our care trajectories, treatment escalation decision-making must account for pre-morbid status, which may, if possible, be best assessed out with crisis situations and acute deterioration. To incorporate patient preferences, completion of formal TEPs in primary care would enable patients who might be too acutely unwell on admission to hospital, to participate in such discussions (of particular relevance to trajectories 1&4). Although it is impossible to anticipate the catastrophic events that occurred for individuals in trajectory 1, it is contended that those individuals who have significant co-morbidities and resulting pre-morbid dependencies (such as those in trajectory 4)

For peer review only - http://bmjopen.bmj.com/site/about/guidelines.xhtml

should be party to sensitive discussion and documentation of a TEP in primary and community care settings.

In summary, this review has highlighted a number of clinically relevant findings, with resulting recommendations, which the authors contend might represent best practice:

- Accurate history taking surrounding premorbid functional status, comorbidity and level of dependency is vital for establishing ceilings of care
- Regular senior clinician involvement results in ongoing review of prognosis and facilitates effective decision-making in complex patients where there is significant clinical uncertainty
- Awareness of a patient's premorbid wishes and where possible, discussion with the patient, should be a priority in deciding ceilings of care
- Discussion with family around prognosis should complement discussions with the patient
- "Fresh eyes" are a valuable tool for reassessing patients' prognosis and should be used more widely for complex patients with significant clinical uncertainty, not responding to treatment
- A senior clinician with overall responsibility for the patient should facilitate multidisciplinary discussion of patients with multiple team involvement
- Earlier involvement of palliative care specialists in patient assessment would aid decision-making and recognition of those who are at the end of life
- Formal TEPs do not preclude active management of reversible conditions, but would aid decision-making and need to be introduced and adopted by clinical teams
- Patients with TEPs need these to be readily visible to teams providing ongoing care to ensure they are followed

CONCLUSION

This review highlighted the complex care management and related decision-making processes of individuals who face acute pathophysiological deterioration leading to death in hospital. Such decision-making processes involve multiple layers of clinicians, from numerous specialities, within often hierarchical teams. Families were involved in contributing to decision-making, in these circumstances patients themselves were frequently too acutely unwell to contribute to all stages of the process. The review identified the need for visibility and clarity of management plans, in spite of the surrounding frame of clinical uncertainty. Even where clear plans were documented they could be buried by subsequent pages of notes, with no effective signposting, a particular problem when further deterioration occurred out-of-hours. Therefore, the review suggests that there is a clear role for formal TEPs to be introduced more widely into routine practice. Opportunities need to be created for individuals to be able to ask for such plans to be made, in consultation with clinicians who know them best, out with the circumstances of acute deterioration.

ACKNOWLEDGEMENTS

The authors would like to thank Dr Marion Penn for her assistance with stratification of the sample. The authors would also like to thank Mrs Sally Dace for her patient and public focused involvement, and the expert clinicians who reviewed the analysis and contributed to the overall interpretation of the data.

AUTHOR CONTRIBUTIONS

NC, AC, SL, MM, CRM, NWP and AR designed the review. NC applied for the necessary approvals. NC and AC extracted the data. NC and AC analysed the data with assistance from SL, NWP and AR. NC and AC drafted the manuscript with assistance from SL, MM, CRM, NWP and AR. All authors

For peer review only - http://bmjopen.bmj.com/site/about/guidelines.xhtml

critically reviewed the manuscript for intellectual content and approved the final version of the paper.

FUNDING

This work was supported by the National Institute for Health Research Collaboration for Leadership in Applied Health Research and Care (NIHR CLAHRC) Wessex which is a partnership between Wessex NHS organisations and partners, including the NIHR Southampton Biomedical Research Centre, and the University of Southampton. Funders had no role in study design, data collection and analysis, decision to publish or preparation of the manuscript.

DISCLAIMER

The views expressed are those of the author(s) and not necessarily those of the NHS, the NIHR or the Department of Health and Social Care.

COMPETING INTERESTS

The authors declare no competing interests.

AVAILABILITY OF DATA

The datasets generated and analysed during this study are not available due to the nature of approval for the study. Support under section 251 of the NHS Act (2006) was obtained via the Health Research Authority's Confidentiality Advisory Group as access to patient identifiable data was required without consent, therefore no additional data can be made available.

For peer review only

REFERENCES

1. Bassford, C (2017) Decisions regarding admission to the ICU and international initiatives to improve the decision-making process. *Critical Care*, 21 (1). 174.
2. Murray, S.A., Kendall, M., Boyd, K. & Sheikh, A., (2005). Illness trajectories and palliative care. *British Medical Journal*, *330*(7498), p.1007.
3. Etkind, S.N., Karno, J., Edmonds, P.M., Carey, I. & Murtagh, F.E., (2014). Supporting patients with uncertain recovery: the use of the AMBER care bundle in an acute hospital. *BMJ Supportive & Palliative Care*, pp.bmjspcare-2013.
4. Higginson, I.J., Rumble, C., Shipman, C., Koffman, J., Sleeman, K.E., Morgan, M., Hopkins, P., Noble, J., Bernal, W., Leonard, S. & Dampier, O., (2016). The value of uncertainty in critical illness? An ethnographic study of patterns and conflicts in care and decision-making trajectories. *BMC Anesthesiology*, *16*(1), p.11.
5. Obolensky, L., Clark, T., Matthew, G. & Mercer, M., (2010). A patient and relative centred evaluation of treatment escalation plans: a replacement for the do-not-resuscitate process. *Journal of Medical Ethics*, *36*(9), pp.518-520.
6. Fritz, Z., Malyon, A., Frankau, J.M., Parker, R.A., Cohn, S., Laroche, C.M., Palmer, C.R. & Fuld, J.P., (2013). The Universal Form of Treatment Options (UFTO) as an alternative to Do Not Attempt Cardiopulmonary Resuscitation (DNACPR) orders: a mixed methods evaluation of the effects on clinical practice and patient care. *PLoS One*, *8*(9), p.e70977.
7. Clements, M., Fuld, J. & Fritz, Z., (2014). Documentation of resuscitation decision-making: a survey of practice in the United Kingdom. *Resuscitation*, *85*(5), pp.606-611.
8. Fritz, Z. & Fuld, J.P., (2015). Development of the Universal Form of Treatment Options (UFTO) as an alternative to Do Not Attempt Cardiopulmonary Resuscitation (DNACPR) orders: a cross-disciplinary approach. *Journal of Evaluation in Clinical Practice*, *21*(1), pp.109-117.
9. Hickman, S.E., Keevern, E. & Hammes, B.J., (2015). Use of the Physician Orders for Life-Sustaining Treatment Program in the Clinical Setting: A Systematic Review of the Literature. *Journal of the American Geriatrics Society*, *63*(2), pp.341-350.
10. Cummings, A., Lund, S., Campling, N., May, C.R., Richardson, A. & Myall, M., (2017). Implementing communication and decision-making interventions directed at goals of care: a theory-led scoping review. *BMJ Open*, *7*(10), p.e017056.
11. Hsieh, H.-F. & S.E. Shannon, (2005). Three approaches to qualitative content analysis. *Qualitative Health Research*, 2005. 15(9): p. 1277-1288.
12. Trebble, T.M., Hansi, N., Hydes, T., Smith, M.A. & Baker, M., (2010). Process mapping the patient journey through health care: an introduction. *British Medical Journal*, *341*(7769), pp.394-397.
13. National Health Services Act (2006). Chapter 41. London: The Stationery Office.
14. Fritz, Z.B.M., Heywood, R.M., Moffat, S.C., Bradshaw, L.E. & Fuld, J.P., (2014). Characteristics and outcome of patients with DNACPR orders in an acute hospital; an observational study. *Resuscitation*, *85*(1), pp.104-108.
15. Hickman, S.E., Nelson, C.A., Moss, A.H., Hammes, B.J., Terwilliger, A., Jackson, A. & Tolle, S.W., (2009). Use of the Physician Orders for Life-Sustaining Treatment (POLST) paradigm program in the hospice setting. *Journal of Palliative Medicine*, *12*(2), pp.133-141.
16. Morris, M., Briant, L., Chidgey-Clark, J., Shouls, S., Carey, I., Hopper, A. & Robinson, C., (2011). Bringing in care planning conversations for patients whose recovery is uncertain: learning from the AMBER care bundle. *BMJ Supportive & Palliative Care*, *1*(1), pp.72-72.
17. Obolensky L, & Mercer M. (2007) Patients' Experiences of Treatment Escalation Plans. *The Intensive Care Society Annual Spring Meeting.*

For peer review only - http://bmjopen.bmj.com/site/about/guidelines.xhtml

18. Brimblecombe, C., Crosbie, D., Lim, W.K. & Hayes, B., (2014). The Goals of Patient Care project: implementing a proactive approach to patient-centred decision-making. *Internal Medicine Journal*, *44*(10), pp.961-966.
19. Newport, K.B., Patel, S., Lyckholm, L., Bobb, B., Coyne, P. and Smith, T.J., 2010. The "PSOST": providers' signout for scope of treatment. *Journal of Palliative Medicine*, *13*(9), pp.1055-1058.

For peer review only

For peer review only - http://bmjopen.bmj.com/site/about/guidelines.xhtml

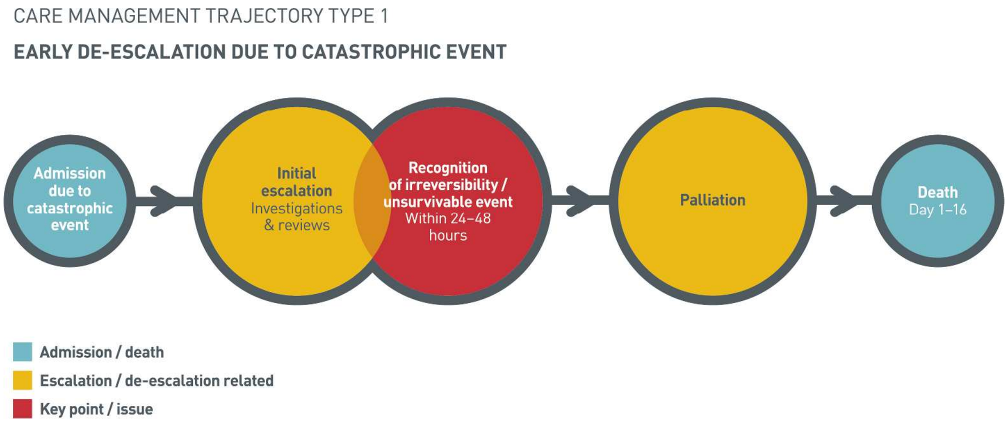

Early De-Escalation Due to Catastrophic Event

264x108mm (300 x 300 DPI)

For peer review only - http://bmjopen.bmj.com/site/about/guidelines.xhtml

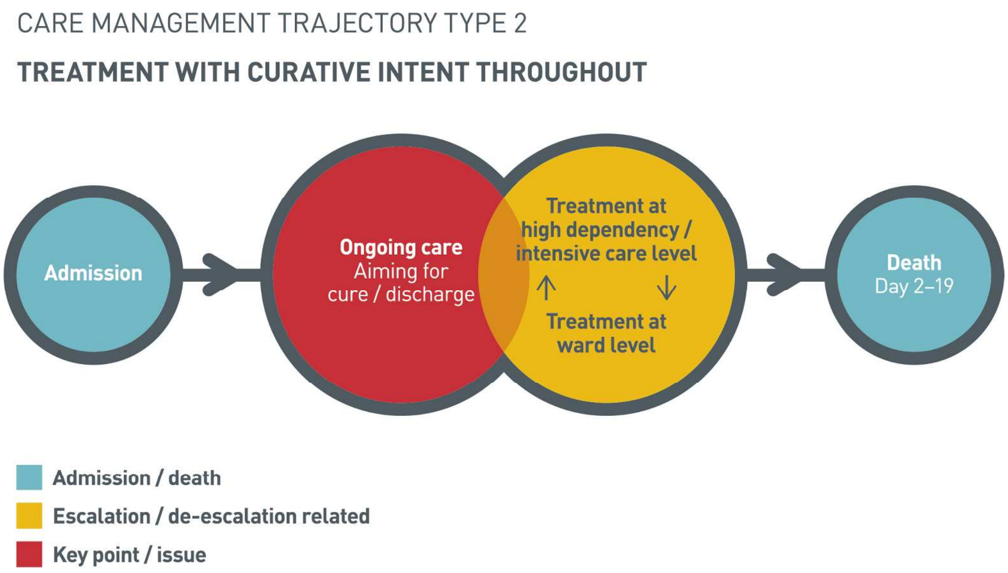

Treatment with Curative Intent Throughout

195x108mm (300 x 300 DPI)

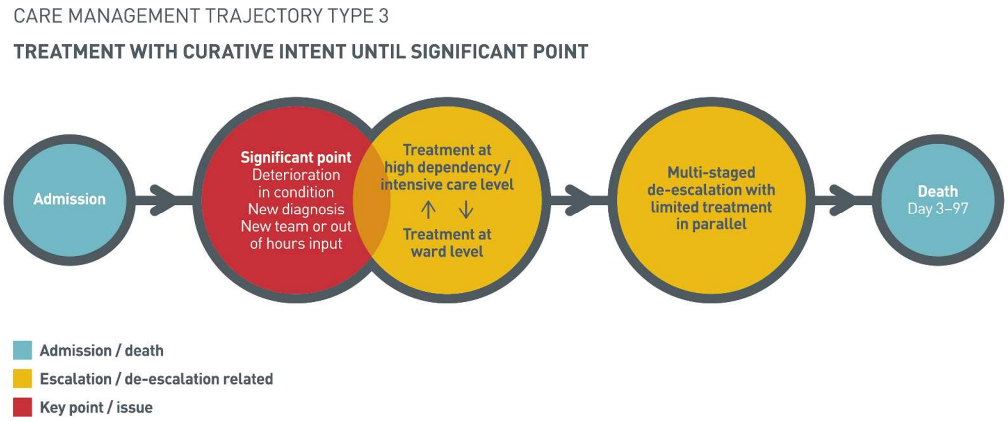

Treatment with Curative Intent until Significant Point

265x108mm (300 x 300 DPI)

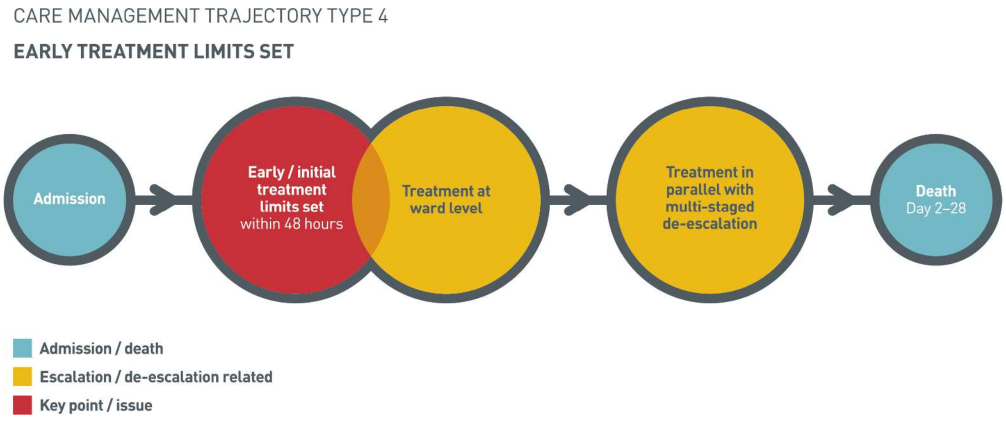

Early Treatment Limits Set

265x108mm (300 x 300 DPI)

For peer review only

Data Extraction Form

**ID number**	
**Age**	
**Gender**	
**Admitting specialty**	
**Date of admission**	
**Date of death**	
**Co-morbidities (from past medical history admission clerking)**	
**History of presenting admission**	
**Cause of death (1a)**	
**Bottom line cause of death (1b or 1c)**	
**Functional status**	
**Potentially avoidable EOLC admission?**	
**Failed EOLC discharge (from evidence in notes)?**	
**Referred to coroner**	
**EOLC pathway used (which and date commenced)?**	
**Was CPR attempted (date)?**	
**Presence of DNACPR (date, signed by, reasons for)?**	
**Is there reference to an advanced care plan?**	

**Date**	Time	Event leading to decision/ discussion (including details surrounding event)	Who recognised/responded to (or led the) event	Speciality (of individual who recognised/responded to event)	Action	Detail about action	Outcome (escalation)	Additional detail around escalation outcome	Outcome (de-escalation)	Additional detail around de-escalation outcome	Involvement of patient and family	Were preferences of patient known in advance?	Detail of discussion with patient/family, including how preferences of others (patient, family, professionals) were taken into account	Details of ward move	Comments
**Line break denotes new episode of deterioration**															

For peer review only

Exemplar Case Studies for the Trajectories[1]

Trajectory 1 Exemplar Case	***Trajectory 2 Exemplar Case***
A 78 year old woman was admitted with reduced consciousness and right sided weakness. She had a past medical history of atrial fibrillation and hypertension, and lived with her daughter. On admission to the emergency department, a chest x-ray, ECG and brain CT were performed, and IV fluids commenced. The decision for a ward-based ceiling of care and DNACPR order was made and subsequently discussed with her daughter, before she was transferred to the acute stroke unit. The following day she was reviewed by the stroke consultant. She showed no signs of improvement; her coma scores remained low and she had clinical signs consistent with a large left middle cerebral artery stroke, which was confirmed by brain CT imaging. Her consultant and clinical team felt that she was unlikely to have any meaningful recovery from her significant brain injury, and the priority of future care should be comfort. A discussion with her family outlined the severity of the stroke, explaining that her condition had not improved in the past 24 hours and that she was unlikely to survive this event. Her family understood the situation and agreed with a palliative approach to care, the consultant emphasizing that she would be kept comfortable. Following this family discussion, a care plan for end of life care was developed and as required palliative medications prescribed. All blood tests, IV fluids and early warning of deterioration scoring ceased. She died peacefully the following day with her family present.	A 76 year old widower, living alone, was admitted due to recent episodes of severe chest pain (both the previous night and reoccurring on the morning of the admission). He had a self-reported history of well controlled non-insulin dependent diabetes. On arrival in accident and emergency he had continued pain, vomiting and ST-segment elevation on ECG. Following consultation with the cardiology consultant and review of the ECG, antiplatelet medications were given and he was taken to the cardiac catheter laboratory for immediate angioplasty. Balloon angioplasty revealed a myocardial infarction, with moderate triple vessel disease. Following angioplasty the patient was transferred to coronary care high dependency, with the aim of discharge after 72 hours if mobilising and pain free.<br><br>On day 2 the patient mobilised with physiotherapy input and was transferred to the cardiac ward. A repeat bedside echocardiogram revealed good left ventricular function. Day 3 the patient continued to improve and discharge was planned for the following day, supported by his family. In the early hours of day 4 an arrest call was made. Cardiopulmonary resuscitation was performed for a suspected pulmonary embolism but the patient remained in asystole throughout and after 30 minutes a consensus decision by the treating clinicians was made to stop.

eview only

Key:

DNACPR – do not attempt cardiopulmonary resuscitation
CCF - congestive cardiac failure
COPD – chronic obstructive pulmonary disease
CT – computerised tomography
ECG - electrocardiogram
IV - intravenous

[1] To ensure confidentiality and anonymity details have been changed and the exemplars generated using features across cases within each type

For peer review only - http://bmjopen.bmj.com/site/about/guidelines.xhtml

Trajectory 3 Exemplar Case	***Trajectory 4 Exemplar Case***
A 79 year old man was admitted with hip pain and reduced mobility following a fall. He had a past medical history of prostate cancer (treated with a radical prostatectomy over 5 years ago), non-insulin dependent diabetes mellitus, chronic kidney disease, ischaemic heart disease, asthma and depression. He was independently mobile with a frame and received carer visits twice daily at home.<br><br>On admission to the emergency department hip X-ray, blood tests and ECG were performed, and IV fluids commenced. His left leg was shortened and externally rotated, and X-rays confirmed a fractured neck of femur. A hemiarthroplasty was performed the following day and he returned to the ward under the care of the orthogeriatric team.<br><br>Over the following days, he received treatment (IV fluids, diuretics and catheterisation) for acute kidney injury and fluid balance issues, as well as ongoing physiotherapy. On day 13 of admission he became tachycardic and hypotensive, triggering an early warning score and review by his medical team. Investigations including a chest X-ray, dipstick urinalysis, ECG and blood tests revealed a likely urinary tract infection, and oral antibiotics were commenced. After 12 hours with no improvement, his antibiotics were switched to IV route and IV fluids were re-commenced. Despite a further 12 hours of antibiotics, his condition continued to deteriorate with spiking fevers and increasing inflammatory markers, reduced urine output and hypotension. Blood cultures were sent, and a decision was made at this point, conveyed to his family, that he should not be for resuscitation or high dependency/intensive care, due to his significant deterioration despite treatment, and his multiple comorbidities.<br><br>Over the weekend, early warning scores prompted junior doctor reviews and a switch of IV antibiotics. Despite this, the patient was agitated and restless. After review by the on-call registrar, it was felt that he should receive symptomatic treatment only, as despite over 72 hours of IV antibiotics he continued to deteriorate, and was now showing signs of distress. A phone call was made to his next of kin to explain that despite treatment, he had progressively deteriorated due to urinary sepsis, his next of kin agreed that supportive care and symptom control were in his best interests and agreed with a DNACPR decision.<br><br>All unnecessary medications, blood tests and observations were ceased. A referral was made to the palliative care team and symptomatic palliative care medications prescribed. He died two days later.	An 88 year old care home resident was admitted to hospital due to an unwitnessed fall during which a head injury was sustained (whilst on warfarin). He had a previous admission to another hospital within the last month also due to a fall, where his diuretic dose was reduced. On admission he had a variable level of consciousness and hypotension. His nursing home stated that he was normally coherent but had experienced a month of reduced eating and drinking. His co-morbidities were noted as CCF, angina, atrial fibrillation, COPD and dementia. His presenting diagnoses were thought to be due to an infective exacerbation of COPD (hospital acquired pneumonia due to the previous admission) and acute kidney injury due to his poor oral intake over the last month.<br><br>Within 24 hours of admission a DNACPR order was signed by the consultant, in consultation with the patient's son due to the underlying CCF and advanced frailty. It was also decided that treatment should occur at ward level, with intubation and ventilation not being appropriate. Over the next couple of days brain CT scan revealed no cause for the reduced consciousness and he improved clinically with first line IV antibiotics and therapy input from physiotherapy and dietetics.<br><br>By day 6 his delirium continued to improve but there were continuing discussions with his son to highlight that although his father had responded to treatment of the infection the underlying conditions meant that he was unlikely to return to the care home. On days seven and eight activation of early warning scores demonstrated low blood pressure, raised respiratory effort and low oxygen saturation levels. Blood tests and a chest X-ray demonstrated left lung consolidation. The IV antibiotics were switched.<br><br>Days eight and nine saw a fall in the consciousness level of the patient despite treatment. The son was called and it was explained that despite treatment his father had developed multi-organ failure. It was agreed that an individualised end of life care plan should be developed focusing on palliation (regular medications and vital signs recordings were stopped) and a referral was made to the palliative care team. On day 10 this team reviewed the patient who was unresponsive but comfortable and pain free. Palliative care medications were administered as required and the patient died with family by his side 14 days after admission.

Summary of Comments from Expert Clinician Review

**Generic**	• The median ages show that we are dealing with a generally elderly population, but not exclusively<br>• The data show that these patients have a lot of co-morbidity<br>• Multiple team involvement emphasised<br>• Clinician hierarchy is important<br>• Decision-making is much more difficult out-of-hours, e.g. between a Consultant who knows the patient/family/case and an on-call Consultant who does not<br>• The more experienced you become as a clinician, the more you recognise uncertainty. The increased exposure to the "unexpected" (in relation to unexpected outcomes of continuing or withdrawing treatment) means that uncertainty remains high<br>• Time limited escalation decisions (e.g. 24-48hrs of ventilation then stop) are more complex and clinicians do not always stick to them. Ceiling of care decisions (e.g. for non-invasive ventilation/not for ventilation) are less complex<br>• Unexpected deterioration will always occur and is impossible to plan for<br>• Lack of escalation related planning usually due to lack of time, lack of "engagement" (with family) and lack of senior re-evaluation of patients over admission course. There are often differences in opinion regarding the reversibility of issues, and differences in opinion regarding pre-admission co-morbidities (and particularly with next of kin)<br>• Increasing culture of "unrealistic" patient/family expectations. These are rare, but can steer decision-making, it is often the more "distant" family members who are not involved all the way through care
**Trajectory 1**	• The catastrophic event occurs at home prior to admission (in contrast to trajectory 3 where the significant point occurs in the hospital)<br>• Typically represents patients with intracranial bleeds, however there is still some uncertainty. Over time with medical developments, the goalposts move with these types of patients
**Trajectory 2**	• There may be 2 subsets of this type: a – those for whom everything is done, but they still die; b – who improve and then there is an event which catches them (e.g. fall, pneumonia)<br>• Does the cardiopulmonary resuscitation reflect less adequate decision-making?
**Trajectory 3**	• The most common trajectory seen in hospital. De-escalation is staged and there may be "bargaining" with families e.g. not everything that team wished has been achieved as a result of an intervention, therefore may agree to continue with status quo for a further 48 hours for example. Deterioration in current condition – the significant point here is greyer and it is harder to make decisions when considering patients "stuck" on high levels of treatment<br>• Missed opportunities in having de-escalation discussion. Frequent continuation of IV fluids and antibiotics with end of life care, as well as a lack of recognition of the dying phase. Due to so many teams being involved and multiple clinicians, so many differences in opinion/views as to when to discuss<br>• New clinical team/out-of-hours input – this is the most uncomfortable in terms of a "trigger"<br>• Outreach teams often see "insidious decline" that the primary care team do not always recognise
**Trajectory 4**	• Significant amount on on-going investigations/treatment despite early limits (ward level care and DNACPR)<br>• The group that would benefit most from formalised TEPs, as potentially the conversations could be had prior to them being admitted to hospital, either in the GP's surgery, care home or hospital outpatients department

For peer review only

**Recommendations for practice**	• Whole cultural shift needed so that individuals are not frightened to talk about what happens when we get ill. Need to create opportunities for discussion outside of crisis situations<br>• Initial discussion with patients/family at or soon after the point of admission is best practice and important in setting expectations in all trajectories<br>• Triggers for escalation related decision-making should be: admission, first senior review, first review by "usual" clinician (if relevant) and any point of deterioration<br>• Earlier and definitive decision-making required (especially trajectory 3 - new clinical team or out-of-hours input as trigger), but decision-making must be accurate, therefore re-evaluation by seniors is key<br>• Significant dependency (especially pertinent to trajectory 4) is important to capture in notes and history taking, should be a trigger for a formalised TEP<br>• Visiting teams may initiate discussions and decisions but these should be implemented by the team with "ownership" who ideally know the patient best<br>• Need for clarity of decisions and what these mean at a practical level e.g. treatment within the ward environment with IV fluids and antibiotics etc.<br>• Need for clarity of terminology and meaning in practice. Palliation means different things to different people and is often a source of confusion. Palliative treatment and full escalation including CPR and ITU are not mutually exclusive. "Ward-based care" is used frequently, meaning potentially aggressive treatment up to the limits of what is possible in a non-HDU and ITU setting (and DNACPR) but it can be misinterpreted in practice as effectively meaning end of life care

review only

Standards for Reporting Qualitative Research (SRQR)*

http://www.equator-network.org/reporting-guidelines/srqr/

Page/line no(s).

Title and abstract

**Title** - Concise description of the nature and topic of the study Identifying the study as qualitative or indicating the approach (e.g., ethnography, grounded theory) or data collection methods (e.g., interview, focus group) is recommended	P1
**Abstract** - Summary of key elements of the study using the abstract format of the intended publication; typically includes background, purpose, methods, results, and conclusions	P2

Introduction

**Problem formulation** - Description and significance of the problem/phenomenon studied; review of relevant theory and empirical work; problem statement	P2-3
**Purpose or research question** - Purpose of the study and specific objectives or questions	P3-4

Methods

**Qualitative approach and research paradigm** - Qualitative approach (e.g., ethnography, grounded theory, case study, phenomenology, narrative research) and guiding theory if appropriate; identifying the research paradigm (e.g., postpositivist, constructivist/ interpretivist) is also recommended; rationale**	P5
**Researcher characteristics and reflexivity** - Researchers' characteristics that may influence the research, including personal attributes, qualifications/experience, relationship with participants, assumptions, and/or presuppositions; potential or actual interaction between researchers' characteristics and the research questions, approach, methods, results, and/or transferability	N/A
**Context** - Setting/site and salient contextual factors; rationale**	P4
**Sampling strategy** - How and why research participants, documents, or events were selected; criteria for deciding when no further sampling was necessary (e.g., sampling saturation); rationale**	P4
**Ethical issues pertaining to human subjects** - Documentation of approval by an appropriate ethics review board and participant consent, or explanation for lack thereof; other confidentiality and data security issues	P6
**Data collection methods** - Types of data collected; details of data collection procedures including (as appropriate) start and stop dates of data collection and analysis, iterative process, triangulation of sources/methods, and modification of procedures in response to evolving study findings; rationale**	P6-7

For peer review only - http://bmjopen.bmj.com/site/about/guidelines.xhtml

**Data collection instruments and technologies** - Description of instruments (e.g., interview guides, questionnaires) and devices (e.g., audio recorders) used for data collection; if/how the instrument(s) changed over the course of the study	P5 + supplementary file 1
**Units of study** - Number and relevant characteristics of participants, documents, or events included in the study; level of participation (could be reported in results)	P6
**Data processing** - Methods for processing data prior to and during analysis, including transcription, data entry, data management and security, verification of data integrity, data coding, and anonymization/de-identification of excerpts	P5-6
**Data analysis** - Process by which inferences, themes, etc., were identified and developed, including the researchers involved in data analysis; usually references a specific paradigm or approach; rationale**	P5-6
**Techniques to enhance trustworthiness** - Techniques to enhance trustworthiness and credibility of data analysis (e.g., member checking, audit trail, triangulation); rationale**	P5-6

Results/findings

**Synthesis and interpretation** - Main findings (e.g., interpretations, inferences, and themes); might include development of a theory or model, or integration with prior research or theory	P6-12
**Links to empirical data** - Evidence (e.g., quotes, field notes, text excerpts, photographs) to substantiate analytic findings	P7-8, p11-12

Discussion

**Integration with prior work, implications, transferability, and contribution(s) to the field -** Short summary of main findings; explanation of how findings and conclusions connect to, support, elaborate on, or challenge conclusions of earlier scholarship; discussion of scope of application/generalizability; identification of unique contribution(s) to scholarship in a discipline or field	P12-14
**Limitations** - Trustworthiness and limitations of findings	P2

Other

**Conflicts of interest** - Potential sources of influence or perceived influence on study conduct and conclusions; how these were managed	P15
**Funding** - Sources of funding and other support; role of funders in data collection, interpretation, and reporting	P14-15

*The authors created the SRQR by searching the literature to identify guidelines, reporting standards, and critical appraisal criteria for qualitative research; reviewing the reference lists of retrieved sources; and contacting experts to gain feedback. The SRQR aims to improve the transparency of all aspects of qualitative research by providing clear standards for reporting qualitative research.

For peer review only - http://bmjopen.bmj.com/site/about/guidelines.xhtml

**The rationale should briefly discuss the justification for choosing that theory, approach, method, or technique rather than other options available, the assumptions and limitations implicit in those choices, and how those choices influence study conclusions and transferability. As appropriate, the rationale for several items might be discussed together.

Reference:

O'Brien BC, Harris IB, Beckman TJ, Reed DA, Cook DA. **Standards for reporting qualitative research: a synthesis of recommendations.** *Academic Medicine*, Vol. 89, No. 9 / Sept 2014 DOI: 10.1097/ACM.0000000000000388

For peer review only

For peer review only - http://bmjopen.bmj.com/site/about/guidelines.xhtml

ESCALATION RELATED DECISION-MAKING IN ACUTE DETERIORATION: A RETROSPECTIVE CASE NOTE REVIEW

Journal:	*BMJ Open*
Manuscript ID	bmjopen-2018-022021.R1
Article Type:	Research
Date Submitted by the Author:	02-May-2018

Complete List of Authors:	Campling, Natasha; University of Southampton Faculty of Health Sciences,<br>Cummings, Amanda; University of Southampton, Faculty of Health Sciences<br>Myall, Michelle; University of Southampton, Faculty of Health Sciences<br>Lund, Susi; University of Southampton, Fcaulty of Health Sciences<br>May, Carl; London School of Hygiene and Tropical Medicine Faculty of Public Health and Policy<br>Pearce, Neil; University Hospital Southampton NHS Foundation Trust<br>Richardson, Alison; University of Southampton, Faculty of Health Sciences

**Primary Subject Heading**:	Health services research
Secondary Subject Heading:	Medical management
Keywords:	decision-making, treatment escalation, de-escalation, palliation, resuscitation, goals of care

SCHOLARONE™
Manuscripts

For peer review only - http://bmjopen.bmj.com/site/about/guidelines.xhtml

ESCALATION RELATED DECISION-MAKING IN ACUTE DETERIORATION: A RETROSPECTIVE CASE NOTE REVIEW

Natasha Campling[1,2], Amanda Cummings[1,2], Michelle Myall[1,2], Susi Lund[1,2], Carl R. May[1,2,3], Neil W. Pearce[3], Alison Richardson[1,2,3]

1. Faculty of Health Sciences, University of Southampton, UK
2. NIHR CLAHRC Wessex, University of Southampton, UK
3. University Hospital Southampton NHS Foundation Trust, Southampton, UK

Corresponding author:
Dr Natasha Campling, Faculty of Health Sciences, University of Southampton, Building 67, University Road, Highfield, Southampton, SO17 1BJ
Tel: +44 (0)23 8059 8228; Fax +44 (0)23 8059 8308; Email: n.c.campling@soton.ac.uk

Key words:
Decision-making; treatment escalation; de-escalation; palliation; resuscitation; goals of care

Word count: 4,966 (excluding abstract, references and tables)

For peer review only - http://bmjopen.bmj.com/site/about/guidelines.xhtml

ABSTRACT (252 words)

Aim: To describe how decision-making interrelates with the sequence of events in individuals who die during admission; and identify situations where formal treatment escalation plans (TEPs) may have utility.

Design and methods: A retrospective case note review, utilising stratified sampling. Two data analysis methods were applied concurrently: directed content analysis; and care management process mapping via annotated timelines for each case. Analysis was followed by expert clinician review (n=7), contributing to data interpretation.

Sample: 45 cases, age range 38-96 years, 23 females and 22 males. Length of admission ranged from <24hours to 97 days.

Results: Process mapping led to a typology of care management, encompassing four trajectories: early de-escalation due to catastrophic event; treatment with curative intent throughout; treatment with curative intent until significant point; and early treatment limits set. Directed content analysis revealed a number of contextual issues influencing decision-making. Three categories were identified: multiple clinician involvement, family involvement, and lack of planning clarity; all framed by clinical complexity and uncertainty.

Conclusions: The review highlighted the complex care management and related decision-making processes for individuals who face acute deterioration. These processes involved multiple clinicians, from numerous specialities, often within hierarchical teams. The review identified the need for visible and clear management plans, in spite of the frame of clinical uncertainty. Formal TEPs can be used to convey such a set of plans. Opportunities need to be created for patients and their families to request these be developed, in consultation with the clinicians who know them best, outside of the traumatic circumstances of acute deterioration.

Strengths and limitations of this study

- There is a lack of description of escalation-related decision-making in the context of deterioration outside of the critical care environment. Our study setting was the comprehensive hospital environment, and individuals who were facing acute deterioration that led to death.
- The study explored clinical decision-making processes: the types and range of decisions made, the involvement of families in these processes, and the interaction between clinical teams. Care management trajectories provoked by acute deterioration were characterised, via typology; including points of significance in the sequence of events. Contextual issues influencing decision-making were described: multiple clinician involvement, family involvement and lack of planning clarity; all framed by clinical complexity and uncertainty.
- While the sample was stratified it was small, selected from a single acute hospital Trust. However two data analysis methods were applied concurrently (followed by expert clinician review): directed content analysis; and care management process mapping via annotated timelines.
- Examination of decision-making processes highlighted areas for improvement and the potential impact of formal treatment escalation plans through pre-emptive decision-making and patient involvement outside of crisis situations.

BACKGROUND

Clinical decision-making in the context of acute deterioration during hospital admission is complex. Such decisions are frequently made in the face of uncertainty, characterised by: lack of underpinning information or diagnostic clarity, necessity for rapid decision-making, and the inability of patients to collaborate in discussions and decisions because of the acuity of their condition [1].

For peer review only

Previous research has focused on the illness trajectories of deteriorating patients, or on clinical decision-making in the specific context of critical care. The wider context of care management and related decision-making remains an un-researched area. Murray et al (2005) highlighted the value of awareness of illness trajectories as a mechanism for clinicians to help plan care to meet patients' needs and for families to cope [2]. More recently, Etkind and colleagues (2014) defined trajectories of final illness among patients who died whilst inpatients [3]. These were defined *a priori* to their case-note review as: predictable (gradual deterioration during admission); predictable (rapid deterioration during admissions); unpredictable course during hospital admission; and sudden death. 149 cases were examined (all deaths over 11 months on five inpatient wards where the AMBER care bundle was implemented) and characterised according to one of four trajectories.

Our study, progresses the above which focused exclusively on illness trajectories, by expanding the focus to care management and understanding of the associated decision-making processes, to inform clinical practice. Higginson and colleagues (2016) explored this area by examining patterns of decision-making, but their work was specific to critical care [4]. Only 16 cases were examined (in combination with interviews and non-participant observation), and four trajectories with different patterns of clinical decision-making identified: curative care from admission (to critical care); oscillating curative and comfort care; shift to comfort care; and comfort care from admission. They emphasised that "conflict" in decision-making could occur, between relatives and staff and between and within clinical teams.

Given the complexity of clinical decision-making related to acuity and deterioration, and perhaps the likelihood for "conflict" [4], there has been a move nationally and internationally to develop and implement formal treatment escalation plans (TEPs). Structured, procedure specific TEPs are proposed as a mechanism by which to improve understanding and communication when escalation related decisions need to be made and acted on [5]. They provide a framework on which to base a conversation and document treatment options that are appropriate if a patient were to become acutely unwell. They vary in both design and use [5,6,7]. Notable examples include: Universal Form of Treatment Options (UFTO) [8], Deciding Right http://www.nescn.nhs.uk/common-themes/deciding-right/ and Physician Orders for Life Sustaining Treatment (POLST) [9]. In the UK, there is growing interest in the national initiative, instigated in 2014, led by the Resuscitation Council and the Royal College of Nursing which generated the Recommended Summary Plan for Emergency Care and Treatment (ReSPECT) http://www.respectprocess.org.uk/

Despite this increasing awareness, little work has examined the implementation of TEPs. This study is part of a wider programme of work to inform the implementation and evaluation of TEPs as part of the *Complexity, Patient Experience and Organisational Behaviour* theme of the NIHR Collaboration for Applied Health Research and Care Wessex (NIHR CLAHRC Wessex). Additionally, the team have undertaken a review of communication and decision-making interventions directed at goals of care, via a theory led scoping review [10].

AIMS/OBJECTIVES

The study explored the care management of those who deteriorate and die during hospital admission, characterising the resources mobilised, in as much detail as could be tracked through recourse to case notes. The aims were: to describe how decision-making processes interrelate with the sequence of events for individuals who die during inpatient admission; and to identify situations where treatment escalation plans may have had utility.

The objectives were:

For peer review only - http://bmjopen.bmj.com/site/about/guidelines.xhtml

1. To identify and characterise, via the generation of a typology, the care management trajectories of hospital inpatients facing acute pathophysiological deterioration which ultimately leads to death.
2. To map clinical decision-making processes, including the involvement of patients and families in decisions, identifying what leads to and triggers changes in management.
3. To identify the potential role of treatment escalation plans in providing a framework to support discussions and recording of decisions.

METHODOLOGY & METHODS

Study Design

A retrospective case note review, exploring the care management of those who die during hospital admission.

Sampling Strategy

The case note review followed an initial audit of death certificate review forms (DCRFs) from all deaths at a single acute hospital Trust in England (n=911) within a six month period (January-July 2015). Case notes of a 5% sample (45 sets of notes) of patients, aged over 18 were reviewed. The DCRF data enabled stratified sampling, ensuring appropriate representation across groups. 32 mutually exclusive strata were created based on whether or not cases had all possible combinations of the following: DNACPR, palliative care team involvement, intensive care/high dependency management, evidence of escalation/de-escalation decision, and unpredictable illness trajectory [3]. Proportionate allocation was used to sample the same fraction from each strata, with a check that the total sample size was calculated as expected (i.e. not affected by rounding of the numbers for each strata to integers).

Data Collection

Data were collected from case notes only. Data collection tracked the period from admission to hospital through to each patient's death to identify: 1) when decisions to escalate or de-escalate treatment were made, 2) how those decisions were made, and 3) who was involved in these decisions. For those with a prolonged admission (> 30 days), data collection was limited to the last 30 days of admission (but included social and clinical data regarding their admission). The following data were extracted:

- Clinical and demographic information regarding admission to hospital, including but not limited to, comorbidities and admitting specialism.
- End of life care and DNACPR information, including but not limited to, whether CPR was attempted.
- Nature of any events leading to a discussion or decision regarding levels of care, who recognised and responded to the event, actions taken, further detail on escalation or de-escalation of care and outcomes from this. Here, "event" referred to episodes such as clinical deterioration, ward rounds, specialist review or emergence of new clinical findings.
- How decisions were documented, including clarity of documentation and use of care plans.
- Evidence of patient and/or family involvement in decision-making and how patient preferences and those of others are taken into account, including whether patient wishes were known in advance.
- Ward movements.
- Date and cause of death.

For peer review only - http://bmjopen.bmj.com/site/about/guidelines.xhtml

Data extraction was undertaken by two clinically qualified researchers (NC & AC) and data recorded using an Excel spreadsheet pro forma (see supplementary file 1). The pro forma was piloted on a set of notes and based on this changes to the form were made to facilitate usability and increase reliability of data extraction. This resulted in: inclusion of all causes of death (not just cause 1a but also underlying causes 1b and 1c), and enabling of free text entry for avoidable end of life care (EOLC) admission and failed EOLC discharge. The revised pro forma was tested by NC and AC on an initial sample of case notes (n=8) to assess utility and consistency of data entry. No further changes were required. At the end of data collection a process of cross-checking by both researchers helped to mitigate against errors, ensure accuracy and consistency.

Data Analysis

Two methods of data analysis were applied concurrently. Firstly, case notes were treated as qualitative data and analysed using directed content analysis [11]. The data within the pro forma were analysed using this method and directed towards: the event leading to the decision or discussion; and the action taken and resulting outcomes, and details regarding involvement and discussion with the patient and family. Data comprised verbatim transcription of relevant entries in the case notes to the pro forma. Additionally, field notes were analysed to capture limitations of case notes as a data source and recurrent issues (sequence of events and triggers for decision-making) across cases.

Secondly, care management process mapping via annotated timelines involving key events were developed for each case [12]. These timelines included: escalation and de-escalation related decisions; involvement of patient and family in decision-making; clinical treatment plans made; investigations undertaken and treatment received; and key clinical information to inform probability of outcomes and prompt decisions.

Timelines were drawn for each case (NC & AC) and then grouped independently by NC & AC (double screened) into one of four care management trajectories which became apparent during analysis. Categorisation of cases by the researchers were compared, with input from two additional clinical members of the research team (SL & AR). Where there was initial disagreement, the pro formas were revisited in a team discussion to agree final categorisation (n=11).

Diagrams were subsequently drawn to represent the group experience of the four care management trajectories. These were iteratively refined (NC, AC, SL & AR). They were combined with tabulated data representing the cases within each trajectory and a case exemplar (case study) and sent to a group of expert clinicians (representing a wide range of specialities) for review. They were asked to consider:

- Do the 4 care management trajectories capture the sequence of events and decision-making processes involved?
- Do the trajectories apply to patients you have seen recently who have then gone on to die whilst in hospital? Could you consider how they do or do not apply?
- Do these data demonstrate potential triggers for decision-making or treatment escalation planning that you would like to see put into practice?
- Is there anything in the data you are surprised by or any other comments you would like to make?

Out of 13 experts approached, 7 commented in detail on the data either face to face or via telephone/email. Their feedback verified: the care management trajectories reflected what

For peer review only - http://bmjopen.bmj.com/site/about/guidelines.xhtml

clinicians encounter in practice and were described in a way they could identify with; the classification of cases to the trajectories; and the authenticity of the case exemplars. They contributed to the overall interpretation of data.

Ethical and Research Governance Considerations

Ethical approval (via the Health Research Authority and Research Ethics Committee South Central – Hampshire A ref: 16/SC/0599) and research governance approval were gained for the study. As access to patient identifiable data (case notes) was required without consent, support under section 251 of the NHS Act (2006) was sought and obtained via the Health Research Authority's Confidentiality Advisory Group [13].

Patient and Public Involvement

A PPI champion worked closely with the research team on this study, and the wider programme, informing all study processes. Involvement led to the recommendation that the team solely access paper based notes (to restrict the amount of data accessed) and not electronic medical records as originally planned.

RESULTS

Sample characteristics

The age range of patients included (in the review of the 45 sets of notes) was 38-96 years, with 23 female and 22 male. The length of admission ranged from <24hours to 97 days. Thirty five patients had a DNACPR in place at time of death. Fifteen patients had palliative care team involvement.

A Typology of Care Management

Analysis via process mapping led to the development of a typology of care management, encompassing four distinct trajectories. The trajectories characterised the sequence of events and decision-making processes through acute pathophysiological deterioration leading to death. They were:

1. Early de-escalation (within 24-48 hours of admission) due to catastrophic event - clinically observable signs and symptoms +/- observable on imaging
2. Treatment with curative intent throughout (no de-escalation) +/- cardiopulmonary resuscitation
3. Treatment with curative intent until significant point
4. Early treatment limits set (within 48 hours of admission)

Table 1 displays the key characteristics of the cases represented by each trajectory. Each care management trajectory is described in sequence below, including a diagrammatic representation of the respective trajectory. Exemplar case studies for each trajectory are included in supplementary file 2. The process of reviewing the data with expert clinicians added a valuable dimension to data interpretation. The depth and range of their feedback, via their experiential knowledge, is summarised in supplementary file 3.

For peer review only - http://bmjopen.bmj.com/site/about/guidelines.xhtml

Table 1 - Key Characteristics of Cases within the Trajectories

TRAJECTORY 1 CASES (n=10)			TRAJECTORY 2 CASES (n=8)		
**AGE (MEDIAN, RANGE)**	79.5 (47-94) years		**AGE (MEDIAN, RANGE)**	83 (53-96) years	
**GENDER**	6 female; 4 male		**GENDER**	1 female; 7 male	
**CACI[1] COMORBIDITIY SCORE (MEDIAN, RANGE)**	5.5 (4-10)		**CACI COMORBIDITIY SCORE (MEDIAN, RANGE)**	6.5 (2-9)	
**SOCIAL HISTORY**	Care/nursing home resident or respite<br>Home carers	2<br>1	**SOCIAL HISTORY**	Care/nursing home resident or respite<br>Home carers	1<br>2
**LENGTH OF ADMISSION (MEDIAN, RANGE)**	3 (1-16[2]) days		**LENGTH OF ADMISSION (MEDIAN, RANGE)**	7.5 (2-19) days	
**PRIMARY REASON FOR ADMISSION**	Cerebrovascular accident<br>Gastrointestinal<br>Sepsis<br>Ischaemic cardiac disease<br>Peripheral vascular disease	3<br>2<br>3<br>1<br>1	**PRIMARY REASON FOR ADMISSION**	Respiratory (infective)<br>Ischaemic/arrhythmic cardiac disease<br>Fall<br>Fracture<br>Cellulitis	3<br>2<br>1<br>1<br>1
**TRIGGERS FOR RECOGNITION OF IRREVERSIBILITY/ UNSURVIVABLE EVENT**<br>*SOME CASES HAD MORE THAN ONE TRIGGER	Imaging results<br>Clinically observable diagnosis<br>Consultant review<br>Reduced consciousness	3<br>4<br>1<br>3	**ONGOING CARE MANAGEMENT/ TREATMENT ISSUES**<br>*ALL CASES HAD MULTIPLE ISSUES	Fluid balance (cardio-renal failure)<br>Acute (on chronic) kidney injury<br>Ischaemic/arrhythmic cardiac disease<br>Respiratory tract infection<br>Urinary tract infection<br>Diabetic control<br>Pulmonary embolism<br>Respiratory failure	4<br>2<br>5<br>8<br>1<br>2<br>1<br>4
**RECEIVED CCO/ITU[3] REVIEW**		1	**RECEIVED CCO/ITU REVIEW**		1
**RECEIVED HDU/ITU[4] CARE**	Intensive care unit	1	**RECEIVED HDU/ITU CARE**	High dependency unit<br>Intensive care unit	1<br>1
			**CPR[5] ATTEMPTED AND UNSUCCESSFUL**		5
			**REASONS FOR NO DE-ESCALATION**<br>*SOME CASES HAD MORE THAN ONE REASON	Awaiting transfer/discharge<br>Patient preference/limited or no family involvement<br>Young/normally fit and well/few comorbidities<br>Post (curative intent) intervention<br>Input from multiple specialist teams	3<br>2<br><br>3<br>1<br>2

For peer review only - http://bmjopen.bmj.com/site/about/guidelines.xhtml

TRAJECTORY 3 CASES (n=18)			TRAJECTORY 4 CASES (n=9)		
**AGE (MEDIAN, RANGE)**	80.5 (38-88) years		**AGE (MEDIAN, RANGE)**	86 (63-91) years	
**GENDER**	12 female; 6 male		**GENDER**	4 female; 5 male	
**CACI COMORBIDITIY SCORE (MEDIAN, RANGE)**	6 (1-12)		**CACI COMORBIDITIY SCORE (MEDIAN, RANGE)**	8 (5-14)	
**SOCIAL HISTORY**	Care/nursing home resident or respite<br>Home carers	1<br>4	**SOCIAL HISTORY**	Care/nursing home resident or respite<br>Home carers	5<br>1
**LENGTH OF ADMISSION (MEDIAN, RANGE)**	17.5 (3-97) days		**LENGTH OF ADMISSION (MEDIAN, RANGE)**	12 (2-28) days	
**PRIMARY REASON FOR ADMISSION**	Gastrointestinal<br>Cerebrovascular accident<br>Respiratory tract infection<br>Urinary tract infection<br>Specialist treatment (chemotherapy, cardio ablation)<br>Haematological<br>Fracture<br>General decline + hypertension<br>Respiratory	3<br>2<br>5<br>1<br>2<br><br>1<br>1<br>2<br>1	**PRIMARY REASON FOR ADMISSION**	Sepsis<br>Respiratory tract infection<br>Malignancy<br>Cerebrovascular accident<br>Fall<br>Acute heart failure	3<br>2<br>1<br>1<br>1<br>1
**RECEIVED CCO/ITU REVIEW**		4	**PRE-EXISITING FACTORS CHARACTERISING ADMISSION**<br>*SOME CASES HAD MORE THAN ONE FACTOR	Frailty<br>History of recent deterioration<br>Pre-existing DNACPR[6]<br>Current malignancy<br>Underlying dementia<br>Already known to palliative care	5<br>2<br>4<br>3<br>3<br>3
**RECEIVED HDU/ITU CARE**	Intensive care unit	3	**PROMPTS FOR SETTING EARLY TREATMENT LIMITS**<br>*SOME CASES HAD MORE THAN ONE PROMPT	Senior clinician review<br>Patient's prior wishes expressed by family<br>Discussion with patient<br>Marked deterioration	7<br>4<br>3<br>4
**SIGNIFICANT POINT TRIGGERING DE-ESCALATION**<br>*SOME CASES HAD MORE THAN ONE TRIGGER	Significant deterioration in current condition<br>New diagnosis leading to deterioration in condition<br>New clinical team/out of hours input recognising poor prognosis	6<br><br>11<br><br>4			

Notes:
[1] CACI - Charlson Age Comorbidity Index www.pmidcalc.org/7722560 (Charlson et al 1994); [2] One individual lived for 16 days despite catastrophic event due to younger age; [3] Critical care outreach/intensive care review; [4] High dependency/intensive care; [5] Cardiopulmonary resuscitation; [6] Do not attempt cardiopulmonary resuscitation

For peer review only - http://bmjopen.bmj.com/site/about/guidelines.xhtml

1. Early De-Escalation Due to Catastrophic Event

This trajectory was characterised by hospital admission due to "catastrophic" events (Figure 1). The event had occurred outside of hospital, was evident at the point of admission, and referenced individuals who were in danger of dying on admission (e.g. patients who were moribund secondary to shock), or those admitted with severe, critical illnesses (e.g. major cerebrovascular accidents).

Following admission, there was a period of initial escalation, with accompanying imaging, diagnostic investigations such as blood tests or electrocardiograms (ECGs), and treatment with intravenous (IV) antibiotics or fluids. This escalation also encompassed senior or specialist (e.g. surgical and intensive care) review.

A key feature of this trajectory was the early (within 24-48 hours) recognition of an unsurvivable or irreversible event. All cases had at least one factor that identified this including: imaging results, clinically observable diagnoses, reduced level of consciousness and/or consultant review. Following recognition of futility, discussions with family and next of kin preceded palliation in all cases bar one. In this case, deterioration and death were so rapid as to prevent timely palliation. This trajectory was generally defined by short admissions, on average, patients died within three days.

Figure 1 – Early de-escalation due to catastrophic event

2. Treatment with Curative Intent Throughout

Trajectory 2 was characterised by treatment with curative intent for the duration of hospital admission (Figure 2). Individuals were admitted with a variety of diagnoses, and admissions were characterised by ongoing care at ward or high dependency/intensive care (HDU/ICU) level for multiple issues. These included fluid balance management associated with cardio-renal failure or acute kidney injury, treatment of infections and management of ischaemic or arrhythmic cardiac disease.

This trajectory was also characterised by the development of new diagnoses (e.g. sepsis) or sudden, unpredictable events (e.g. pulmonary embolism) which ultimately led to death. However, in these cases, such events did not trigger de-escalation (as in trajectory 3), patients were actively treated until death. In 5 of the 8 cases unsuccessful cardiopulmonary resuscitation occurred prior to death. In the three remaining individuals, DNACPR orders had been stimulated by senior clinician reviews and/or family discussions.

The reasons underlying a lack of de-escalation related to patient characteristics, individual preferences and the delivery or focus of health care. Some individuals were younger or normally fit and well with minimal comorbidities, whilst others expressed a preference for active treatment. For some, a recent intervention with curative intent, or the fact that they were awaiting discharge or transfer to alternative settings, meant that de-escalation was not a consideration. In others, the involvement of multiple specialist teams meant that the leading specialism (and thus the team who might be expected to make de-escalation decisions) was not clear.

Figure 2 – Treatment with curative intent throughout

3. Treatment with Curative Intent until Significant Point

Trajectory 3 was characterised by curative intent treatment until a significant point, triggering de-escalation of care (Figure 3). These triggers included significant deterioration in the patient's current

For peer review only - http://bmjopen.bmj.com/site/about/guidelines.xhtml

condition (in the absence of a new diagnosis) for example a reduction in consciousness level or patient agitation/distress, and new diagnoses (e.g. infection or malignancy) which led to deterioration in the patient's condition. A third trigger involved a new clinical specialism or out-of-hours review recognising poor prognosis and the futility of current treatment, prompting de-escalation.

All triggers for de-escalation prompted discussions with next of kin, family and the patient or other clinical teams (if under shared-care management). Following these discussions, multi-staged de-escalation ensued. The first stage involved the setting of ceilings of care and DNACPR orders. This first stage at times occurred prior to family discussion, but such discussion always preceded the second stage, which included stopping vital sign observations, early-warning activation scores and invasive investigations/treatments. In some cases, a time and intensity limited trial of treatment (e.g. antibiotics) preceded a third stage of de-escalation, palliation. For patients receiving HDU/ICU level care, the latter stages of de-escalation involved the withdrawal of treatment. There was usually some degree of treatment provided in parallel to multi-staged de-escalation, although this was limited, typically involving antibiotics and IV fluids. The time between the significant point which triggered de-escalation and patient death was between 0-10 days, however this trajectory was characterised by the longest and most varied admission length, 3-97 days.

Figure 3 – Treatment with curative intent until significant point

4. Early Treatment Limits Set

Trajectory 4 was characterised by the presence of early treatment limits, set within 48 hours of admission (Figure 4). The triggers for setting limits included patient refusal of treatment, discussions with family, senior clinician review and marked deterioration in the patients' condition. Crucially, these triggers occurred against backgrounds of: history of recent deterioration, frailty, underlying diagnoses of dementia or malignancy, and the presence of pre-existing DNACPR orders and palliative care involvement. In line with this, the patients in this trajectory had the highest average comorbidity scores and ages.

Early treatment limits formed the start of a multi-staged de-escalation process, which occurred across the duration of admission. This de-escalation started with treatment limits (DNACPR, not for intubation/dialysis/ICU care, ward based care) before progressing to more active de-escalation (ceasing early warning scores, ceasing antibiotics/IV fluids/regular medications, palliation and commencement of an individualised end of life care plan).

Key to this trajectory was the level of on-going treatment in parallel with the staged de-escalation. Despite early treatment limits being set, on-going treatment involved a far more extensive range of treatment (interventions, therapy and medications) than in trajectory 3. Interventions included catheterisation, nasogastric tubes and blood transfusions. There was therapy input from physiotherapists, speech and language therapists, dieticians and occupational therapy teams, and medications included diuretics and antibiotics. Nonetheless, on-going treatment was restricted to a ward environment as clinical history meant these individuals were not candidates for intensive treatment.

Figure 4 – Early treatment limits set

<u>The Categories</u>

For peer review only - http://bmjopen.bmj.com/site/about/guidelines.xhtml

In addition to the care management typology, our directed content analysis revealed a number of contextual issues, which influenced decision-making. We identified three inter-linked categories consisting of: multiple clinician involvement, family involvement, and lack of planning clarity. The categories were framed by clinical complexity and uncertainty.

Clinical Complexity and Uncertainty

The cases demonstrated clinical complexity caused in the main by multiple co-morbidities, new diagnoses or undiagnosed conditions, and challenging management e.g. of sepsis, kidney injury and frailty. Challenging management of fluid balance issues associated with multiple concurrent comorbidities, and the onset of new infections, were a frequent occurrence. A lack of clarity surrounding definitive diagnoses often meant that clinicians were "working in the dark" trying to maximise management despite ongoing uncertainty. Although there were some more clearly defined diagnoses and management paths evidenced (such as stroke), with greater clinical predictability, these cases were in the minority.

Decision-making was complicated by frequently changing clinical situations, particularly in relation to new findings or diagnoses. Escalation-related decisions were required that could adapt to these changing situations, where previous management plans were rapidly rendered inappropriate.

Multiple Clinician Involvement

Clinical management via multiple specialities, therapy and outreach teams, could preclude sight of the patient's prognosis. This was evidenced by treatment decisions and therapy involvement that did not always reflect an individual's prognosis. Likewise, the practicalities of input from multiple specialisms, including numerous repeat reviews and interplay between different teams, often acted to elongate decision-making processes, and added complexity when no-one team took responsibility for leading decisions.

There was evidence of a hierarchy in decision-making, with senior clinicians most often instigating decisions. Junior doctors were less likely to make escalation related decisions, especially concerning placing limitations on, or removal of, treatments. Junior doctors, when required to make decisions alone (particularly those working out-of-hours) were more likely to continue treatment escalation, especially in the absence of pre-specified escalation plans. As such, there was a clear role for senior review, with registrars and consultants instigating the majority of decisions regarding treatment limits and withdrawal of treatment.

The transfer of patients between wards and clinical teams added complexity to decision processes. There was evidence of transfers resulting in de-escalation plans being overlooked, however in other circumstances, ward or team moves prompted new reviews and the initiation of appropriate planning. The positive influence of new perspectives or "fresh eyes" on escalation related decision-making was apparent, especially via out-of-hours clinicians. It appeared that individuals not caught up in the day-to-day management of patient care were able to see the "bigger picture" regarding care management, often initiating ceilings of care, or prompting escalation plans.

Family Involvement

The role and influence of the family was often central in the decision-making process. It was apparent that escalation-related decisions (i.e. whether to continue to increase the intensiveness of treatment e.g. dialysis, intubation and ventilation or maintain treatment at ward level) were often established and actioned before discussions with the family took place. Whereas, de-escalation

For peer review only - http://bmjopen.bmj.com/site/about/guidelines.xhtml

related decisions (such as ceasing treatments and commencing palliation) were postponed until after discussions with family. Family involvement and consensus agreement always preceded the withdrawal of treatment (e.g. organ support and ventilation). This reflects the moral imperative to discuss such decisions with family. Additionally, family were involved in the decision-making process for DNACPR orders where there was any concern about patient competency. Families also played an important role in providing collateral histories for clinicians, enabling decision-making to be placed in the context of an individual's recent health. This was particularly the case with older patients where families could highlight weeks or months of recent deterioration or recurrent infections, aiding the admission clerking, and facilitating early treatment limits being set (trajectory 4).

The impact of the familial role was most apparent when absent. In a few cases, where patients had limited or no family involvement, or lacked the physical presence of family members to prompt discussions, de-escalation decisions were not made (those in trajectory 2). In contrast, where families were engaged they were frequently involved in consultative decision-making with clinical teams. These families were often able to provide clear instructions to clinicians because of their knowledge of patients' prior wishes. For example, relatives were recorded as stating that the "*patient wouldn't want to live like this*", and were therefore more likely to endorse clinician recommendations for treatment withdrawal. Additionally, families often agreed with recommendations that if the patient did not respond to treatment then a move to focus on palliation should occur. The converse did apply, although only in a few cases, whereby families stated that the patient would "*want all done*". In situations where families were unsure of the patient's wishes, further team meetings with the family were always undertaken.

Lack of Planning Clarity

The data revealed a general lack of clarity and visibility regarding management plans in the case notes. However, the clinical complexity of these cases at times precluded the making of escalation related plans or led to them being held in a type of uncommitted management "status" until certainty was gained. Even where cogent management plans were made, they may not have been followed because there were no effective methods for signposting clinicians to plans buried in subsequent pages of notes. In addition, where management plans involved clear de-escalation, these were not always followed. This was sometimes more than just due to the lack of visibility in the notes, but also due to clinical complexity and unpredictability of deterioration, with fluctuations leading to patients temporarily improving or stabilising.

Initial clerking and history taking was paramount to the quality of decision-making throughout admission. This was particularly apparent where clerking histories appeared "lost", with key factors not carrying through into decisions made. Where an important co-morbidity was not acknowledged during the admission clerking, this could continue to influence care over the length of admission.

DISCUSSION

This case note review and qualitative analysis, identified four care management trajectories, defining and mapping clinical decision-making processes in the context of acute pathophysiological deterioration. All trajectories from admission through to death, were framed by clinical complexity and related uncertainty. In general, such complexity confounded decision-making processes. Nonetheless, in a minority of profoundly complex cases (e.g. older age, associated frailty, comorbid and premorbid statuses), complexity could encourage escalation related decision-making. This was apparent in the fourth trajectory, where early treatment limits were set based on patients' pre-

For peer review only - http://bmjopen.bmj.com/site/about/guidelines.xhtml

admission morbidity. This concurs with Fritz et al (2014) who in a retrospective case note review found a lower threshold for completing DNACPR orders in patients with multiple co-morbidities [14].

The trajectories identified here expand those previously described by Higginson et al (2016) which were exclusive to critical care, as they are applicable to hospital inpatients irrespective of care setting [4]. Consequently, our trajectories highlight a) significant points in care trajectories where senior secondary review and re-evaluation of management plans would be valuable, and b) groups of patients for whom a formal TEP would be of particular benefit, as a framework to support discussions and the recording of decisions.

Our findings display significant points in care management trajectories (1&3). These included the recognition of irreversibility, deterioration in current condition, new diagnoses leading to deterioration, and new clinical or out-of-hours team involvement. It was these points that triggered discussions around escalation, and ultimately decision-making. We propose that whilst acting as triggers, these points in trajectory 3 cases also present missed opportunities, for earlier, timely decision-making. It was frequent for deterioration to occur out-of-hours, with important decisions left to on-call teams and sometimes more junior clinicians. As previous studies have shown, this can preclude decisions that reflect the best-interests and preferences of the patient [12]. Here, clear management plans are required that pre-empt the possibility of deterioration and outline the patients' wishes in such circumstances, as well as realistic parameters of care.

The absence of significant points in some cases by which to trigger decision-making, such as those in trajectory 2, leads to a proposition made by the study's expert clinical reviewers that strategic senior reviews are required. It is possible that earlier senior review secondary to a post-admission review, may enable appropriate re-evaluation and alter management plans. Nevertheless, a lack of recognition of the dying phase, even by senior clinicians, highlighted the role and contribution of palliative care teams in questioning ongoing investigations or treatment, and stimulating appropriate symptom control.

It is known that formal TEPs are helpful in stimulating discussions, formulating clear plans, ensuring patient preferences are considered [5,15], and perceived as a good idea by patients, families and healthcare professionals [5,15,16,17]. In addition, they help healthcare professionals structure their discussions with patients and families, and record their decisions, improving documentation clarity [18] and escalation-related communication within clinical teams [16,19]. Despite this, in the case notes reviewed, there were no recorded instances of a formal TEP being used to aid decision-making. Four patients held pre-existing DNACPR orders, but none had evidence of an advance care plan or formal TEP. Despite the small number of pre-existing DNACPR orders in the review, their existence led clinicians to have wider escalation related discussions with patients and families. There is also a pragmatic argument that documenting a DNACPR decision should trigger consideration of a TEP, as a logical continuation of the resuscitation discussion. However, based on our care trajectories, treatment escalation decision-making must account for pre-morbid status, which may, if possible, be best assessed outside of crisis situations and acute deterioration. To incorporate patient preferences, completion of formal TEPs in primary care would enable patients who might be too acutely unwell on admission to hospital, to participate in such discussions (of particular relevance to trajectories 1&4). Although it is impossible to anticipate the catastrophic events that occurred for individuals in trajectory 1, it is contended that those individuals who have significant co-morbidities and resulting pre-morbid dependencies (such as those in trajectory 4)

For peer review only - http://bmjopen.bmj.com/site/about/guidelines.xhtml

should be party to sensitive discussion and documentation of a TEP in primary and community care settings.

In summary, this review has highlighted a number of clinically relevant findings, with resulting recommendations, which the authors contend might represent best practice:

- Accurate history taking surrounding premorbid functional status, comorbidity and level of dependency is vital for establishing ceilings of care
- Regular senior clinician involvement results in ongoing review of prognosis and facilitates effective decision-making in complex patients where there is significant clinical uncertainty
- Awareness of a patient's premorbid wishes and where possible, discussion with the patient, should be a priority in deciding ceilings of care
- Discussion with family around prognosis should complement discussions with the patient
- "Fresh eyes" are a valuable tool for reassessing patients' prognosis and should be used more widely for complex patients with significant clinical uncertainty, not responding to treatment
- A senior clinician with overall responsibility for the patient should facilitate multidisciplinary discussion of patients with multiple team involvement
- Earlier involvement of palliative care specialists in patient assessment would aid decision-making and recognition of those who are at the end of life
- Formal TEPs do not preclude active management of reversible conditions, but would aid decision-making and need to be introduced and adopted by clinical teams
- Patients with TEPs need these to be readily visible to teams providing ongoing care to ensure they are followed

CONCLUSION

This review highlighted the complex care management and related decision-making processes of individuals who face acute pathophysiological deterioration leading to death in hospital. Such decision-making processes involve multiple layers of clinicians, from numerous specialities, within often hierarchical teams. Families were involved in contributing to decision-making, in these circumstances patients themselves were frequently too acutely unwell to contribute to all stages of the process. The review identified the need for visibility and clarity of management plans, in spite of the surrounding frame of clinical uncertainty. Even where clear plans were documented they could be buried by subsequent pages of notes, with no effective signposting, a particular problem when further deterioration occurred out-of-hours. Therefore, the review suggests that there is a clear role for formal TEPs to be introduced more widely into routine practice. Opportunities need to be created for patients and their families to be able to ask for such plans to be made, in consultation with clinicians who know them best, outside of the circumstances of acute deterioration.

ACKNOWLEDGEMENTS

The authors would like to thank Dr Marion Penn for her assistance with stratification of the sample. The authors would also like to thank Mrs Sally Dace for her patient and public focused involvement, and the expert clinicians who reviewed the analysis and contributed to the overall interpretation of the data.

AUTHOR CONTRIBUTIONS

NC, AC, SL, MM, CRM, NWP and AR designed the review. NC applied for the necessary approvals. NC and AC extracted the data. NC and AC analysed the data with assistance from SL, NWP and AR. NC and AC drafted the manuscript with assistance from SL, MM, CRM, NWP and AR. All authors

For peer review only - http://bmjopen.bmj.com/site/about/guidelines.xhtml

critically reviewed the manuscript for intellectual content and approved the final version of the paper.

FUNDING

This work was supported by the National Institute for Health Research Collaboration for Leadership in Applied Health Research and Care (NIHR CLAHRC) Wessex which is a partnership between Wessex NHS organisations and partners, including the NIHR Southampton Biomedical Research Centre, and the University of Southampton. Funders had no role in study design, data collection and analysis, decision to publish or preparation of the manuscript.

DISCLAIMER

The views expressed are those of the author(s) and not necessarily those of the NHS, the NIHR or the Department of Health and Social Care.

COMPETING INTERESTS

The authors declare no competing interests.

AVAILABILITY OF DATA

The datasets generated and analysed during this study are not available due to the nature of approval for the study. Support under section 251 of the NHS Act (2006) was obtained via the Health Research Authority's Confidentiality Advisory Group as access to patient identifiable data was required without consent, therefore no additional data can be made available.

For peer review only

REFERENCES

1. Bassford, C (2017) Decisions regarding admission to the ICU and international initiatives to improve the decision-making process. *Critical Care*, 21 (1). 174.
2. Murray, S.A., Kendall, M., Boyd, K. & Sheikh, A., (2005). Illness trajectories and palliative care. *British Medical Journal*, *330*(7498), p.1007.
3. Etkind, S.N., Karno, J., Edmonds, P.M., Carey, I. & Murtagh, F.E., (2014). Supporting patients with uncertain recovery: the use of the AMBER care bundle in an acute hospital. *BMJ Supportive & Palliative Care*, pp.bmjspcare-2013.
4. Higginson, I.J., Rumble, C., Shipman, C., Koffman, J., Sleeman, K.E., Morgan, M., Hopkins, P., Noble, J., Bernal, W., Leonard, S. & Dampier, O., (2016). The value of uncertainty in critical illness? An ethnographic study of patterns and conflicts in care and decision-making trajectories. *BMC Anesthesiology*, *16*(1), p.11.
5. Obolensky, L., Clark, T., Matthew, G. & Mercer, M., (2010). A patient and relative centred evaluation of treatment escalation plans: a replacement for the do-not-resuscitate process. *Journal of Medical Ethics*, *36*(9), pp.518-520.
6. Fritz, Z., Malyon, A., Frankau, J.M., Parker, R.A., Cohn, S., Laroche, C.M., Palmer, C.R. & Fuld, J.P., (2013). The Universal Form of Treatment Options (UFTO) as an alternative to Do Not Attempt Cardiopulmonary Resuscitation (DNACPR) orders: a mixed methods evaluation of the effects on clinical practice and patient care. *PLoS One*, *8*(9), p.e70977.
7. Clements, M., Fuld, J. & Fritz, Z., (2014). Documentation of resuscitation decision-making: a survey of practice in the United Kingdom. *Resuscitation*, *85*(5), pp.606-611.
8. Fritz, Z. & Fuld, J.P., (2015). Development of the Universal Form of Treatment Options (UFTO) as an alternative to Do Not Attempt Cardiopulmonary Resuscitation (DNACPR) orders: a cross-disciplinary approach. *Journal of Evaluation in Clinical Practice*, *21*(1), pp.109-117.
9. Hickman, S.E., Keevern, E. & Hammes, B.J., (2015). Use of the Physician Orders for Life-Sustaining Treatment Program in the Clinical Setting: A Systematic Review of the Literature. *Journal of the American Geriatrics Society*, *63*(2), pp.341-350.
10. Cummings, A., Lund, S., Campling, N., May, C.R., Richardson, A. & Myall, M., (2017). Implementing communication and decision-making interventions directed at goals of care: a theory-led scoping review. *BMJ Open*, *7*(10), p.e017056.
11. Hsieh, H.-F. & S.E. Shannon, (2005). Three approaches to qualitative content analysis. *Qualitative Health Research*, 2005. 15(9): p. 1277-1288.
12. Trebble, T.M., Hansi, N., Hydes, T., Smith, M.A. & Baker, M., (2010). Process mapping the patient journey through health care: an introduction. *British Medical Journal*, *341*(7769), pp.394-397.
13. National Health Services Act (2006). Chapter 41. London: The Stationery Office.
14. Fritz, Z.B.M., Heywood, R.M., Moffat, S.C., Bradshaw, L.E. & Fuld, J.P., (2014). Characteristics and outcome of patients with DNACPR orders in an acute hospital; an observational study. *Resuscitation*, *85*(1), pp.104-108.
15. Hickman, S.E., Nelson, C.A., Moss, A.H., Hammes, B.J., Terwilliger, A., Jackson, A. & Tolle, S.W., (2009). Use of the Physician Orders for Life-Sustaining Treatment (POLST) paradigm program in the hospice setting. *Journal of Palliative Medicine*, *12*(2), pp.133-141.
16. Morris, M., Briant, L., Chidgey-Clark, J., Shouls, S., Carey, I., Hopper, A. & Robinson, C., (2011). Bringing in care planning conversations for patients whose recovery is uncertain: learning from the AMBER care bundle. *BMJ Supportive & Palliative Care*, *1*(1), pp.72-72.
17. Obolensky L, & Mercer M. (2007) Patients' Experiences of Treatment Escalation Plans. *The Intensive Care Society Annual Spring Meeting.*

For peer review only - http://bmjopen.bmj.com/site/about/guidelines.xhtml

18. Brimblecombe, C., Crosbie, D., Lim, W.K. & Hayes, B., (2014). The Goals of Patient Care project: implementing a proactive approach to patient-centred decision-making. *Internal Medicine Journal*, *44*(10), pp.961-966.
19. Newport, K.B., Patel, S., Lyckholm, L., Bobb, B., Coyne, P. and Smith, T.J., 2010. The “PSOST”: providers' signout for scope of treatment. *Journal of Palliative Medicine*, *13*(9), pp.1055-1058.

For peer review only

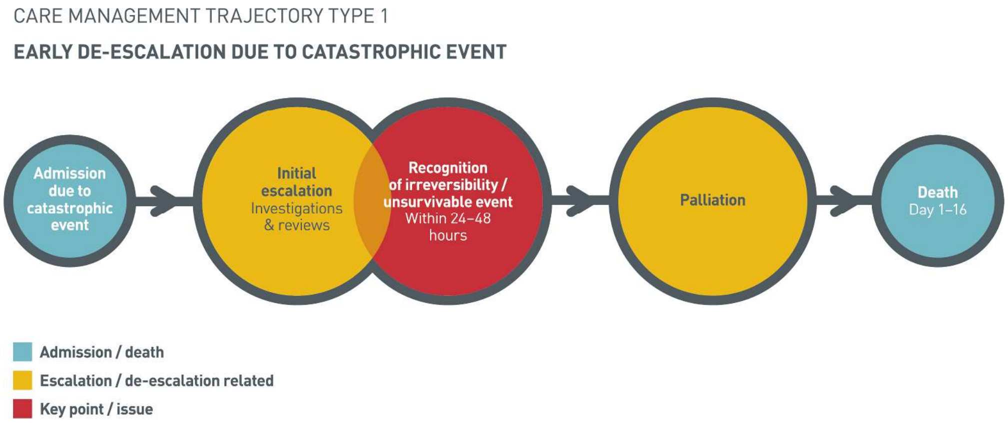

Early De-Escalation Due to Catastrophic Event

264x108mm (300 x 300 DPI)

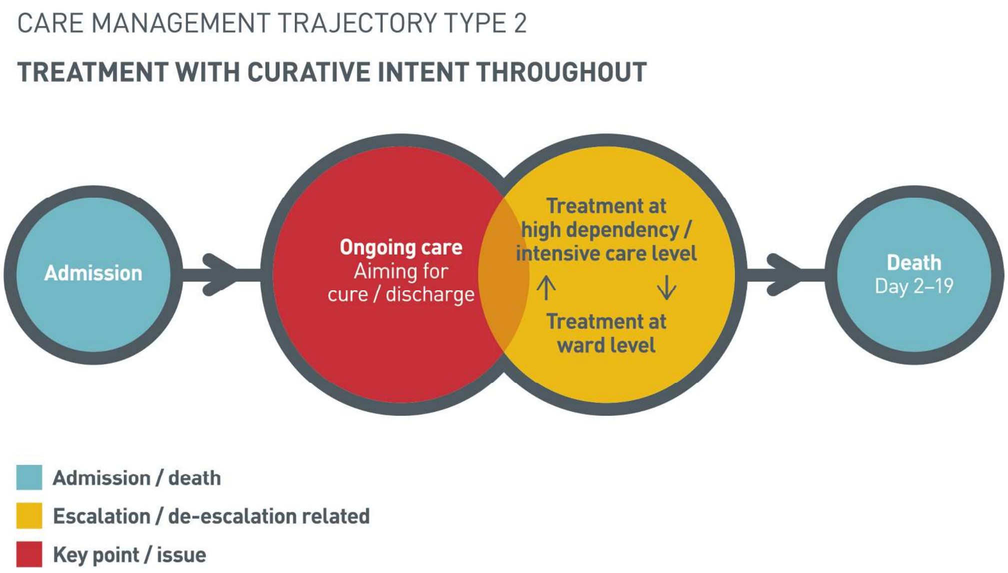

Treatment with Curative Intent Throughout

195x108mm (300 x 300 DPI)

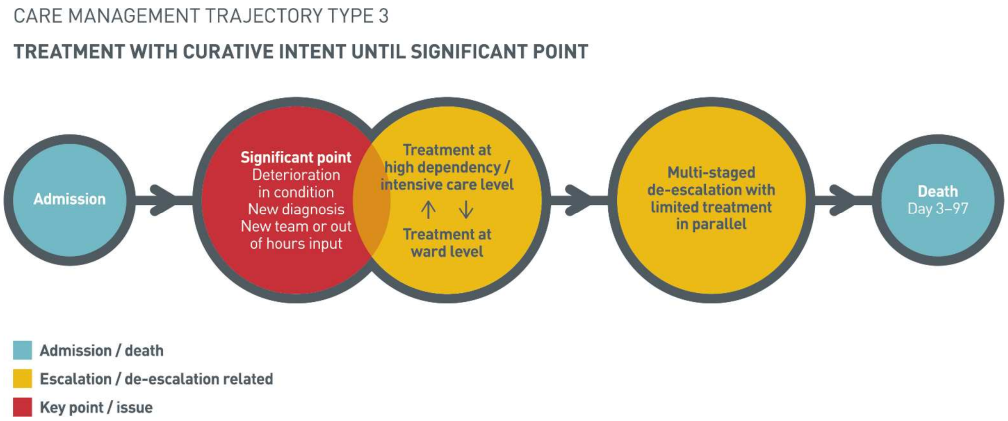

Treatment with Curative Intent until Significant Point

265x108mm (300 x 300 DPI)

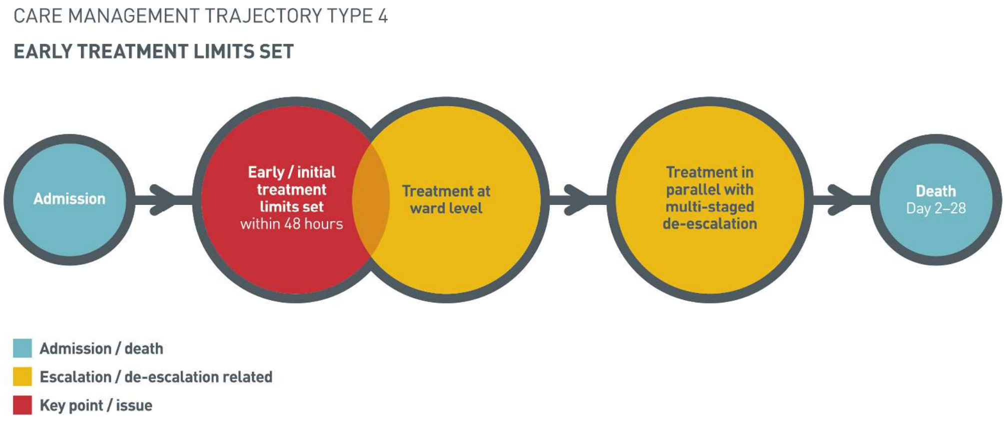

Early Treatment Limits Set

265x108mm (300 x 300 DPI)

Data Extraction Form

**ID number**	
**Age**	
**Gender**	
**Admitting specialty**	
**Date of admission**	
**Date of death**	
**Co-morbidities (from past medical history admission clerking)**	
**History of presenting admission**	
**Cause of death (1a)**	
**Bottom line cause of death (1b or 1c)**	
**Functional status**	
**Potentially avoidable EOLC admission?**	
**Failed EOLC discharge (from evidence in notes)?**	
**Referred to coroner**	
**EOLC pathway used (which and date commenced)?**	
**Was CPR attempted (date)?**	
**Presence of DNACPR (date, signed by, reasons for)?**	
**Is there reference to an advanced care plan?**	

**Date**	Time	Event leading to decision/ discussion (including details surrounding event)	Who recognised/responded to (or led the) event	Speciality (of individual who recognised/responded to event)	Action	Detail about action	Outcome (escalation)	Additional detail around escalation outcome	Outcome (de-escalation)	Additional detail around de-escalation outcome	Involvement of patient and family	Were preferences of patient known in advance?	Detail of discussion with patient/family, including how preferences of others (patient, family, professionals) were taken into account	Details of ward move	Comments
**Line break denotes new episode of deterioration**															

For peer review only - http://bmjopen.bmj.com/site/about/guidelines.xhtml

Exemplar Case Studies for the Trajectories[1]

Trajectory 1 Exemplar Case	*Trajectory 2 Exemplar Case*
A 78 year old woman was admitted with reduced consciousness and right sided weakness. She had a past medical history of atrial fibrillation and hypertension, and lived with her daughter. On admission to the emergency department, a chest x-ray, ECG and brain CT were performed, and IV fluids commenced. The decision for a ward-based ceiling of care and DNACPR order was made and subsequently discussed with her daughter, before she was transferred to the acute stroke unit. The following day she was reviewed by the stroke consultant. She showed no signs of improvement; her coma scores remained low and she had clinical signs consistent with a large left middle cerebral artery stroke, which was confirmed by brain CT imaging. Her consultant and clinical team felt that she was unlikely to have any meaningful recovery from her significant brain injury, and the priority of future care should be comfort. A discussion with her family outlined the severity of the stroke, explaining that her condition had not improved in the past 24 hours and that she was unlikely to survive this event. Her family understood the situation and agreed with a palliative approach to care, the consultant emphasizing that she would be kept comfortable. Following this family discussion, a care plan for end of life care was developed and as required palliative medications prescribed. All blood tests, IV fluids and early warning of deterioration scoring ceased. She died peacefully the following day with her family present.	A 76 year old widower, living alone, was admitted due to recent episodes of severe chest pain (both the previous night and reoccurring on the morning of the admission). He had a self-reported history of well controlled non-insulin dependent diabetes. On arrival in accident and emergency he had continued pain, vomiting and ST-segment elevation on ECG. Following consultation with the cardiology consultant and review of the ECG, antiplatelet medications were given and he was taken to the cardiac catheter laboratory for immediate angioplasty. Balloon angioplasty revealed a myocardial infarction, with moderate triple vessel disease. Following angioplasty the patient was transferred to coronary care high dependency, with the aim of discharge after 72 hours if mobilising and pain free.<br><br>On day 2 the patient mobilised with physiotherapy input and was transferred to the cardiac ward. A repeat bedside echocardiogram revealed good left ventricular function. Day 3 the patient continued to improve and discharge was planned for the following day, supported by his family. In the early hours of day 4 an arrest call was made. Cardiopulmonary resuscitation was performed for a suspected pulmonary embolism but the patient remained in asystole throughout and after 30 minutes a consensus decision by the treating clinicians was made to stop.

Key:
DNACPR – do not attempt cardiopulmonary resuscitation
CCF - congestive cardiac failure
COPD – chronic obstructive pulmonary disease
CT – computerised tomography
ECG - electrocardiogram
IV - intravenous

[1] To ensure confidentiality and anonymity details have been changed and the exemplars generated using features across cases within each type

For peer review only - http://bmjopen.bmj.com/site/about/guidelines.xhtml

Trajectory 3 Exemplar Case	*Trajectory 4 Exemplar Case*
A 79 year old man was admitted with hip pain and reduced mobility following a fall. He had a past medical history of prostate cancer (treated with a radical prostatectomy over 5 years ago), non-insulin dependent diabetes mellitus, chronic kidney disease, ischaemic heart disease, asthma and depression. He was independently mobile with a frame and received carer visits twice daily at home.<br><br>On admission to the emergency department hip X-ray, blood tests and ECG were performed, and IV fluids commenced. His left leg was shortened and externally rotated, and X-rays confirmed a fractured neck of femur. A hemiarthroplasty was performed the following day and he returned to the ward under the care of the orthogeriatric team.<br><br>Over the following days, he received treatment (IV fluids, diuretics and catheterisation) for acute kidney injury and fluid balance issues, as well as ongoing physiotherapy. On day 13 of admission he became tachycardic and hypotensive, triggering an early warning score and review by his medical team. Investigations including a chest X-ray, dipstick urinalysis, ECG and blood tests revealed a likely urinary tract infection, and oral antibiotics were commenced. After 12 hours with no improvement, his antibiotics were switched to IV route and IV fluids were re-commenced. Despite a further 12 hours of antibiotics, his condition continued to deteriorate with spiking fevers and increasing inflammatory markers, reduced urine output and hypotension. Blood cultures were sent, and a decision was made at this point, conveyed to his family, that he should not be for resuscitation or high dependency/intensive care, due to his significant deterioration despite treatment, and his multiple comorbidities.<br><br>Over the weekend, early warning scores prompted junior doctor reviews and a switch of IV antibiotics. Despite this, the patient was agitated and restless. After review by the on-call registrar, it was felt that he should receive symptomatic treatment only, as despite over 72 hours of IV antibiotics he continued to deteriorate, and was now showing signs of distress. A phone call was made to his next of kin to explain that despite treatment, he had progressively deteriorated due to urinary sepsis, his next of kin agreed that supportive care and symptom control were in his best interests and agreed with a DNACPR decision.<br><br>All unnecessary medications, blood tests and observations were ceased. A referral was made to the palliative care team and symptomatic palliative care medications prescribed. He died two days later.	An 88 year old care home resident was admitted to hospital due to an unwitnessed fall during which a head injury was sustained (whilst on warfarin). He had a previous admission to another hospital within the last month also due to a fall, where his diuretic dose was reduced. On admission he had a variable level of consciousness and hypotension. His nursing home stated that he was normally coherent but had experienced a month of reduced eating and drinking. His co-morbidities were noted as CCF, angina, atrial fibrillation, COPD and dementia. His presenting diagnoses were thought to be due to an infective exacerbation of COPD (hospital acquired pneumonia due to the previous admission) and acute kidney injury due to his poor oral intake over the last month.<br><br>Within 24 hours of admission a DNACPR order was signed by the consultant, in consultation with the patient's son due to the underlying CCF and advanced frailty. It was also decided that treatment should occur at ward level, with intubation and ventilation not being appropriate. Over the next couple of days brain CT scan revealed no cause for the reduced consciousness and he improved clinically with first line IV antibiotics and therapy input from physiotherapy and dietetics.<br><br>By day 6 his delirium continued to improve but there were continuing discussions with his son to highlight that although his father had responded to treatment of the infection the underlying conditions meant that he was unlikely to return to the care home. On days seven and eight activation of early warning scores demonstrated low blood pressure, raised respiratory effort and low oxygen saturation levels. Blood tests and a chest X-ray demonstrated left lung consolidation. The IV antibiotics were switched.<br><br>Days eight and nine saw a fall in the consciousness level of the patient despite treatment. The son was called and it was explained that despite treatment his father had developed multi-organ failure. It was agreed that an individualised end of life care plan should be developed focusing on palliation (regular medications and vital signs recordings were stopped) and a referral was made to the palliative care team. On day 10 this team reviewed the patient who was unresponsive but comfortable and pain free. Palliative care medications were administered as required and the patient died with family by his side 14 days after admission.

Summary of Comments from Expert Clinician Review

**Generic**	• The median ages show that we are dealing with a generally elderly population, but not exclusively<br>• The data show that these patients have a lot of co-morbidity<br>• Multiple team involvement emphasised<br>• Clinician hierarchy is important<br>• Decision-making is much more difficult out-of-hours, e.g. between a Consultant who knows the patient/family/case and an on-call Consultant who does not<br>• The more experienced you become as a clinician, the more you recognise uncertainty. The increased exposure to the "unexpected" (in relation to unexpected outcomes of continuing or withdrawing treatment) means that uncertainty remains high<br>• Time limited escalation decisions (e.g. 24-48hrs of ventilation then stop) are more complex and clinicians do not always stick to them. Ceiling of care decisions (e.g. for non-invasive ventilation/not for ventilation) are less complex<br>• Unexpected deterioration will always occur and is impossible to plan for<br>• Lack of escalation related planning usually due to lack of time, lack of "engagement" (with family) and lack of senior re-evaluation of patients over admission course. There are often differences in opinion regarding the reversibility of issues, and differences in opinion regarding pre-admission co-morbidities (and particularly with next of kin)<br>• Increasing culture of "unrealistic" patient/family expectations. These are rare, but can steer decision-making, it is often the more "distant" family members who are not involved all the way through care
**Trajectory 1**	• The catastrophic event occurs at home prior to admission (in contrast to trajectory 3 where the significant point occurs in the hospital)<br>• Typically represents patients with intracranial bleeds, however there is still some uncertainty. Over time with medical developments, the goalposts move with these types of patients
**Trajectory 2**	• There may be 2 subsets of this type: a – those for whom everything is done, but they still die; b – who improve and then there is an event which catches them (e.g. fall, pneumonia)<br>• Does the cardiopulmonary resuscitation reflect less adequate decision-making?
**Trajectory 3**	• The most common trajectory seen in hospital. De-escalation is staged and there may be "bargaining" with families e.g. not everything that team wished has been achieved as a result of an intervention, therefore may agree to continue with status quo for a further 48 hours for example. Deterioration in current condition – the significant point here is greyer and it is harder to make decisions when considering patients "stuck" on high levels of treatment<br>• Missed opportunities in having de-escalation discussion. Frequent continuation of IV fluids and antibiotics with end of life care, as well as a lack of recognition of the dying phase. Due to so many teams being involved and multiple clinicians, so many differences in opinion/views as to when to discuss<br>• New clinical team/out-of-hours input – this is the most uncomfortable in terms of a "trigger"<br>• Outreach teams often see "insidious decline" that the primary care team do not always recognise
**Trajectory 4**	• Significant amount on on-going investigations/treatment despite early limits (ward level care and DNACPR)<br>• The group that would benefit most from formalised TEPs, as potentially the conversations could be had prior to them being admitted to hospital, either in the GP's surgery, care home or hospital outpatients department

For peer review only

**Recommendations for practice**	• Whole cultural shift needed so that individuals are not frightened to talk about what happens when we get ill. Need to create opportunities for discussion outside of crisis situations<br>• Initial discussion with patients/family at or soon after the point of admission is best practice and important in setting expectations in all trajectories<br>• Triggers for escalation related decision-making should be: admission, first senior review, first review by "usual" clinician (if relevant) and any point of deterioration<br>• Earlier and definitive decision-making required (especially trajectory 3 - new clinical team or out-of-hours input as trigger), but decision-making must be accurate, therefore re-evaluation by seniors is key<br>• Significant dependency (especially pertinent to trajectory 4) is important to capture in notes and history taking, should be a trigger for a formalised TEP<br>• Visiting teams may initiate discussions and decisions but these should be implemented by the team with "ownership" who ideally know the patient best<br>• Need for clarity of decisions and what these mean at a practical level e.g. treatment within the ward environment with IV fluids and antibiotics etc.<br>• Need for clarity of terminology and meaning in practice. Palliation means different things to different people and is often a source of confusion. Palliative treatment and full escalation including CPR and ITU are not mutually exclusive. "Ward-based care" is used frequently, meaning potentially aggressive treatment up to the limits of what is possible in a non-HDU and ITU setting (and DNACPR) but it can be misinterpreted in practice as effectively meaning end of life care

review only

Standards for Reporting Qualitative Research (SRQR)*

http://www.equator-network.org/reporting-guidelines/srqr/

Title and abstract

	Page/line no(s).
**Title** - Concise description of the nature and topic of the study Identifying the study as qualitative or indicating the approach (e.g., ethnography, grounded theory) or data collection methods (e.g., interview, focus group) is recommended	P1
**Abstract** - Summary of key elements of the study using the abstract format of the intended publication; typically includes background, purpose, methods, results, and conclusions	P2

Introduction

**Problem formulation** - Description and significance of the problem/phenomenon studied; review of relevant theory and empirical work; problem statement	P2-3
**Purpose or research question** - Purpose of the study and specific objectives or questions	P3-4

Methods

**Qualitative approach and research paradigm** - Qualitative approach (e.g., ethnography, grounded theory, case study, phenomenology, narrative research) and guiding theory if appropriate; identifying the research paradigm (e.g., postpositivist, constructivist/ interpretivist) is also recommended; rationale**	P5
**Researcher characteristics and reflexivity** - Researchers' characteristics that may influence the research, including personal attributes, qualifications/experience, relationship with participants, assumptions, and/or presuppositions; potential or actual interaction between researchers' characteristics and the research questions, approach, methods, results, and/or transferability	N/A
**Context** - Setting/site and salient contextual factors; rationale**	P4
**Sampling strategy** - How and why research participants, documents, or events were selected; criteria for deciding when no further sampling was necessary (e.g., sampling saturation); rationale**	P4
**Ethical issues pertaining to human subjects** - Documentation of approval by an appropriate ethics review board and participant consent, or explanation for lack thereof; other confidentiality and data security issues	P6
**Data collection methods** - Types of data collected; details of data collection procedures including (as appropriate) start and stop dates of data collection and analysis, iterative process, triangulation of sources/methods, and modification of procedures in response to evolving study findings; rationale**	P6-7

For peer review only - http://bmjopen.bmj.com/site/about/guidelines.xhtml

For peer review only

**Data collection instruments and technologies** - Description of instruments (e.g., interview guides, questionnaires) and devices (e.g., audio recorders) used for data collection; if/how the instrument(s) changed over the course of the study	P5 + supplementary file 1
**Units of study** - Number and relevant characteristics of participants, documents, or events included in the study; level of participation (could be reported in results)	P6
**Data processing** - Methods for processing data prior to and during analysis, including transcription, data entry, data management and security, verification of data integrity, data coding, and anonymization/de-identification of excerpts	P5-6
**Data analysis** - Process by which inferences, themes, etc., were identified and developed, including the researchers involved in data analysis; usually references a specific paradigm or approach; rationale**	P5-6
**Techniques to enhance trustworthiness** - Techniques to enhance trustworthiness and credibility of data analysis (e.g., member checking, audit trail, triangulation); rationale**	P5-6

Results/findings

**Synthesis and interpretation** - Main findings (e.g., interpretations, inferences, and themes); might include development of a theory or model, or integration with prior research or theory	P6-12
**Links to empirical data** - Evidence (e.g., quotes, field notes, text excerpts, photographs) to substantiate analytic findings	P7-8, p11-12

Discussion

**Integration with prior work, implications, transferability, and contribution(s) to the field -** Short summary of main findings; explanation of how findings and conclusions connect to, support, elaborate on, or challenge conclusions of earlier scholarship; discussion of scope of application/generalizability; identification of unique contribution(s) to scholarship in a discipline or field	P12-14
**Limitations** - Trustworthiness and limitations of findings	P2

Other

**Conflicts of interest** - Potential sources of influence or perceived influence on study conduct and conclusions; how these were managed	P15
**Funding** - Sources of funding and other support; role of funders in data collection, interpretation, and reporting	P14-15

*The authors created the SRQR by searching the literature to identify guidelines, reporting standards, and critical appraisal criteria for qualitative research; reviewing the reference lists of retrieved sources; and contacting experts to gain feedback. The SRQR aims to improve the transparency of all aspects of qualitative research by providing clear standards for reporting qualitative research.

For peer review only - http://bmjopen.bmj.com/site/about/guidelines.xhtml

**The rationale should briefly discuss the justification for choosing that theory, approach, method, or technique rather than other options available, the assumptions and limitations implicit in those choices, and how those choices influence study conclusions and transferability. As appropriate, the rationale for several items might be discussed together.

Reference:

O'Brien BC, Harris IB, Beckman TJ, Reed DA, Cook DA. **Standards for reporting qualitative research: a synthesis of recommendations.** *Academic Medicine*, Vol. 89, No. 9 / Sept 2014 DOI: 10.1097/ACM.0000000000000388

For peer review only

For peer review only - http://bmjopen.bmj.com/site/about/guidelines.xhtml
